# Divergent Bacterial Communities in Water and Sediment of Chlorinated Drinking Water Storage Tanks

**DOI:** 10.1021/acsestwater.5c00928

**Published:** 2025-12-11

**Authors:** Eva Bridges, Sienna Bircher, Kara Cunningham, Vinila Vasam, John Hando, Emily Garner

**Affiliations:** † Wadsworth Department of Civil & Environmental Engineering, 5631 West Virginia University, Morgantown, West Virginia 26506, United States; ‡ Department of Industrial and Management Systems Engineering, West Virginia University, Morgantown, West Virginia 26506, United States; § School of Natural Resources and the Environment, West Virginia University, Morgantown, West Virginia 26506, United States

**Keywords:** drinking water distribution system, storage tanks, bacterial community, microbial community

## Abstract

Properly operated and maintained drinking water distribution system (DWDS) storage tanks are crucial for allocating safe drinking water, but varying operational processes and infrequent maintenance can result in water quality degradation, including disinfectant residual loss, sediment accumulation, bacterial growth, and potential contamination. This study assessed how the physical, chemical, and hydraulic characteristics of representative chlorinated DWDS tanks relate to bacterial communities in water and sediment; investigated water quality variation by depth within tanks; and explored the infrastructure and management characteristics influencing bacterial community composition in tanks. Bulk water and sediment samples were collected from seven tanks in a chlorinated DWDS system, and 16S rRNA gene amplicon sequencing was used to characterize the bacterial community. Bulk water and sediment communities were distinct, dominated by Alphaproteobacteria and Gammaproteobacteria, respectively. Spatial variations as a function of distance from the treatment plant, tank-specific characteristics, and sediment accumulation were found to shape bacterial communities within tanks. Total coliforms and *Escherichia coli* were undetectable in all water samples, but genetic signatures indicated the presence of multiple genera associated with opportunistic pathogens (OPs). This study aims to establish a deeper understanding of the bacterial community within DWDS tanks and the impact that tank conditions and characteristics have on DWDS water quality.

## Graphical abstract

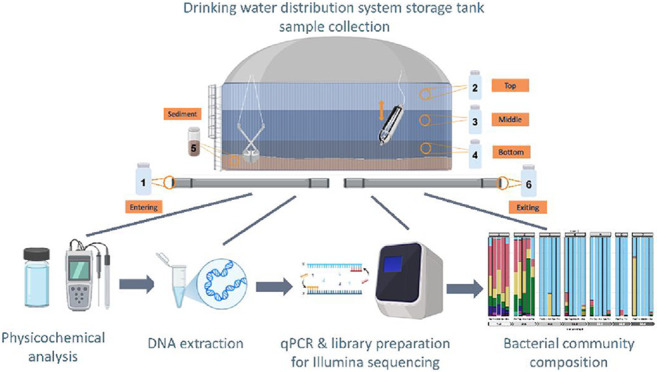

## 1 Introduction

Drinking water distribution systems (DWDSs) store, transport, and deliver water to the public, but water quality can fluctuate and degrade as it travels from the treatment plant to consumers’ taps.^1,2^ Storage tanks are a vital component of this infrastructure and can include elevated storage tanks, standpipes, ground-level tanks, and underground reservoirs.^3^ Properly operated and maintained DWDS storage tanks play a crucial role in supplying high-quality, finished drinking water to serviced areas, equalizing system pressure, providing emergency response usage, and managing diurnal fluctuations in user demand.^3–5^ However, prolonged water storage can lead to increased water age, which is associated with water quality degradation, posing heightened risks to public health.^6^

Several factors can influence the quality of water stored in DWDS tanks: disinfectant residual type and levels,^7,8^ microbial growth and sediment accumulation,^3,5,9,10^ turnover rates and stagnation,^4,11,12^ location within the DWDS,^13^ and tank size and material.^12^ These conditions vary across and within DWDSs due to differences in source water quality, fire flow requirements, environmental factors, treatment processes, system construction and operation, and system size.^11,14^

Aging DWDS infrastructure may negatively impact water quality through the intrusion of debris, sediment, and contaminants via access points including hatches, sidewall joints, vents, and overflow piping.^3^ Corrosion, influenced by the DWDS infrastructure material, results in the release of particles that accumulate as sediment in storage tanks. Substantial accumulation of sediment in DWDS storage tanks has been observed, with one tank documented to have as much as 28 in. of sediment after 15 years without cleaning.^3^

The sediment that accumulates in storage tanks has important implications for the presence of both pathogens and disinfection byproducts (DBPs), as it creates an environment suitable for bacterial growth, increased demand for disinfectant residual, and reduced aesthetic qualities.^3,9,15–17^ Factors such as water stagnation, elevated temperatures, sediment accumulation, and low levels of disinfectant residual all favor and promote the regrowth of microbes in storage tanks.^18^ Although there is limited knowledge about biofilm presence and growth on tank sediments, previous studies have highlighted their significance by documenting microbes, including opportunistic pathogens (OPs), in accumulated sediments.^10,19–22^ For example, municipal drinking water storage tank sediments from multiple DWDSs have been found to convey genetic signatures for a variety of OPs, including *Legionella* spp., *Mycobacterium* spp., and *Pseudomonas aeruginosa*.^9,23^

The microbial attributes associated with DWDS tank water and sediments remain poorly understood, largely due to difficulties in accessing and sampling storage tanks. Most monitoring occurs at tank inlets and outlets, providing little insight into the conditions throughout tanks. Sediment samples are typically collected in conjunction with tank cleaning or draining,^9,14,23^ limiting available data. No federal mandates for tank cleaning currently exist in the United States, but the American Water Works Association (AWWA) recommends cleanings and inspections every 3–5 years. However, many systems fail to meet this goal due to high costs and a lack of enforcement.^5,24^ A 2020 Association of State Drinking Water Administrators (ASDWA) survey found that 38 states inspect above-ground-level storage tanks, 22 states recommend tank cleanings, and eight states require it.^25^

Given the limited scope of available knowledge on tanks and the microbial communities associated with the water and sediment therein, the objectives of this study were to (1) assess how the physical, chemical, and hydraulic characteristics of representative chlorinated DWDS storage tanks relate to bacterial communities in water and sediment; (2) investigate the variation in water quality within individual tanks in relation to different collection depths; and (3) identify infrastructure and management characteristics that influence microorganisms in drinking water tanks. Improving our understanding of the bacterial communities within DWDS storage tanks and the impact that tank conditions and characteristics can have on distribution system water quality can inform improved management of these systems and aid in the mitigation of potential public health risks.

## 2 Methods

### 2.1 Study Sites and Sample Collection

The studied utility serves more than 70,000 residents via 450 miles of buried main pipes (44% polyvinyl chloride, 28% asbestos cement) with diameters ranging from 2 to 36 in. and 42 water storage tanks. Source water blended from a river and reservoir is treated through coagulation, flocculation, sedimentation, rapid sand filtration, membrane filtration, and disinfection with free chlorine, where a free chlorine residual is maintained throughout distribution and storage. Sodium tripolyphosphate and lime are added for corrosion control, resulting in an effluent pH of 7.8–8.0.

Seven storage tanks were selected as sampling sites to capture a variety of characteristics based on previous observations from utility personnel (sediment accumulation, DBP formation, and low chlorine residuals) and several parameters of interest (Table 1). The utility practices daily overflowing and periodic flushing at tanks with elevated water age (tanks 6 and 7) to minimize DBPs. Storage tanks are dosed as needed with high-test hypochlorite (∼65% chlorine) during warm and dry periods (June–October) to prevent complete decay of the disinfectant residual.

**Table 1:** Characteristics of the Selected Tanks

tank ID	tank type	diameter × height (ft)	capacity (gal)	material	turnover rate (days)	pipe miles from DWTP	construction date	last cleaning
1	ground-level storage tank	180 × 30	2,000,000	welded steel	0.63	1.4	1961	2023
2	underground reservoir	8 (height)	188,000	concrete	0.43	2.8	1952	none recorded
3	ground-level storage tank	61 × 25	500,000	welded steel	2.06	9.8	2001	none recorded
4	ground-level storage tank	26 × 24	100,000	welded steel	4.00	10.2	1972	none recorded
5	ground-level storage tank	30 × 28	150,000	welded steel	4.66	13.8	unknown; acquired in 1994	2020
6	ground-level storage tank	22 × 30	87,800	glass lined	7.45	15.9	2012	none recorded
7	ground-level storage tank	12 × 13	10,000	welded steel	1.75	18.0	unknown; acquired in 1994	none recorded

Tank water at three depths, tank influent and effluent water, and sediment samples were collected from each tank site in June 2024 (Figure 1). Isolated grab samples of water were collected directly below the tank access hatch via separate sterilized bacon bomb samplers (George Taylor Brass Bronze Works) for each depth throughout the tank (top, middle, and bottom). Samples were collected 1–2 ft below the water surface to represent the top of the tank (Figure 1, bottle 2), the middle of the tank’s water depth (bottle 3), and 1–2 ft above the bottom of the tank (bottle 4) (refer to Text S1 for further details). Only influent, top, effluent, and sediment samples were collected at tank 6 due to the difficulty associated with accessing the tank and sampling equipment.

**Figure 1:**
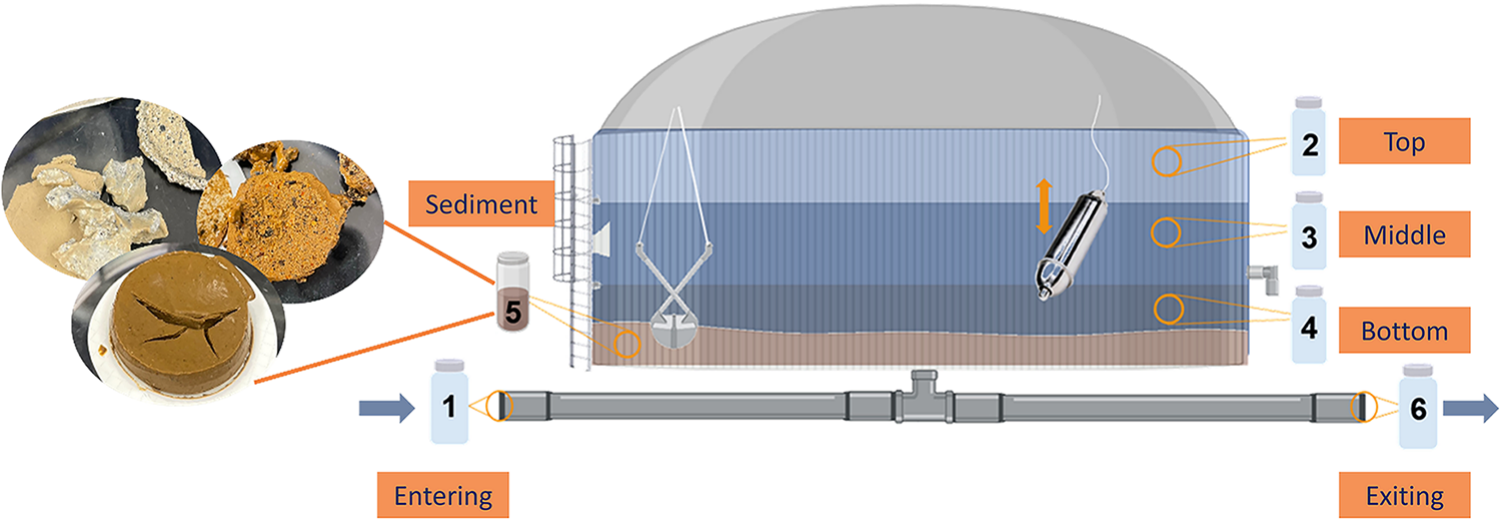
Drinking water storage tank—water and sediment sample collection techniques and locations. Sample depths within the tank are represented by bottles: 1 = entering (Ent), 2 = top, 3 = middle (Mid), 4 = bottom (Bot), 5 = sediment, 6 = exiting (Ext).

Water samples were collected from DWDS locations that represent water quality both before and after tank storage. Prestorage samples, referred to as tank influent or entering samples (bottle 1), and poststorage samples, referred to as tank effluent or exiting samples (bottle 6), were obtained after thorough flushing at hydrants, booster stations, or tank pits, depending on the tank location and surrounding infrastructure.

Bulk water samples were collected for microbiological analysis (autoclaved 3 L polypropylene bottles dosed with 48 mg/L sodium thiosulfate for chlorine quenching), water chemistry analysis (autoclaved 1 L polypropylene bottles), and organic carbon and total trihalomethanes (TTHMs) (250 mL amber glass bottles baked at 550 °C) at each tank site and each sample depth. Samples for TTHM analysis were transferred to triplicate 40 mL vials and acidified with 25 mg of ascorbic acid powder.

Sediment samples were collected via a bacon bomb or dredge (LaMotte) from the bottom of each tank and transferred to an autoclaved 1 L polypropylene bottle (bottle 5). Replicates A and B refer to two different aliquots of sediment samples taken after overall sediment sample homogenization.

Samples were transported to the lab on ice and then stored at 4 °C during processing, which was completed within 7 h of sample collection. Water samples were filter-concentrated onto 0.2 μm mixed cellulose ester filters (MilliporeSigma) for molecular analysis. Filters were torn into ∼1 cm^2^ pieces with sterile tweezers and stored in DNA extraction lysing matrix tubes provided in the commercial kits used for subsequent DNA extraction. Sediment samples were dewatered by decanting liquid from settled samples and then filtering through a 0.45 μm mixed cellulose ester membrane filter (MilliporeSigma). Samples were stored at −20 °C until DNA extraction was performed. Additional details are provided in Text S1.

### 2.2 Physicochemical Water Quality

Various physicochemical water characteristics were analyzed for each collection point, including temperature, pH, conductivity, total and free chlorine, total dissolved solids (TDS), nitrite, nitrate, iron, turbidity, total organic carbon (TOC), and total coliforms and *Escherichia coli* (refer to Text S2 for detailed methods). Preserved TTHM vials were delivered to Pace Analytical (Morgantown, WV) for analysis via EPA Method 524.2.

### 2.3 DNA Extraction

DNA extraction was performed using the Fast DNA SPIN Kit or the Fast DNA SPIN Kit for Soil (MP Biomedicals) for bulk water and sediment samples, respectively. DNA extractions were performed on empty lysing matrix tubes from each kit to provide a DNA extraction blank as well as both filter types to provide filter blanks. DNA extracts were stored at −20 °C.

### 2.4 Quantification of 16S rRNA Genes

Quantitative polymerase chain reaction (qPCR) was performed in triplicate to quantify the universal bacterial 16S rRNA gene and provide an approximation of total bacterial abundance using previously published forward primer 1369F and reverse primer 1492R.^26^ DNA extracts were diluted prior to qPCR, at a 1:2 ratio for water samples and a 1:20 ratio for sediment samples, based on a trial with a subset of samples to minimize inhibition. A matrix spike was used to identify sediment DNA extracts that still exhibited inhibition after dilution; these samples were then further treated with the *OneStep* PCR Inhibitor Removal Kit (Zymo Research). All reactions were run in triplicate, and each 96-well plate included a triplicate negative control of molecular-grade water and a triplicate standard curve of seven serially diluted (10^7^–10^1^ gene copies) target DNA standards. Primers and gBlocks standards were purchased from Integrated DNA Technologies, Inc. The limit of quantification for the 16S rRNA gene was 100 gene copies per reaction, which was the lowest standard that was amplified in triplicate during each run (Table S17). Refer to Text S3 and Tables S1–S3 for further details.

### 2.5 16S rRNA Gene Amplicon Sequencing

Amplicon libraries were prepared using a two-step nested-polymerase chain reaction (PCR) method for low biomass samples, adapted from Shaw et al. and previously applied to DWDS samples by Ferrebee et al.^27,28^ Universal 16S rRNA primers 8F^29^ and 1492R^26^ were used for the first round of PCR. Products were then amplified using barcoded 515F^30^ and 926R^31^ primers, which target the V4–V5 regions of the 16S rRNA gene.

Target amplification was verified by using gel electrophoresis. Amplification products were quantified using a Qubit Fluorometer 2.0 and a dsDNA HS assay kit (Life Technologies). An equal mass of 240 ng of DNA from each sample was pooled and then purified with the Invitrogen PureLink PCR Purification Kit. Additional details are provided in Text S4.

Sequencing was conducted at the Duke Center for Genomic and Computational Biology (Durham, NC) on an Illumina MiSeq using a 250 cycle paired-end protocol. Bacterial community profiling was performed using QIIME2 ^32^ (Quantitative Insights into Microbial Ecology) (version 2021.4), via the West Virginia University (WVU) High Performance Computing facility. Quality filtering was performed using the DADA2 ^33^ plugin to remove low-quality sequences, chimeras, errors, and other nontarget sequences. Operational taxonomic units (OTUs) were clustered based on sequence similarity of >99% and taxonomically classified using a training classifier based on the Silva rRNA database (version 132).^34^ α diversity was calculated using the Shannon diversity index, and β diversity was calculated using Bray–Curtis dissimilarity with a sampling depth equal to the minimum number of reads (14,945).

### 2.6 Characterization of Sediments

Extracellular polymeric substances (EPSs) were extracted to quantify protein and polysaccharide content from sediments and associated biofilms according to previously published methods^35–39^ (refer to Text S5 for detailed methods).

Tank sediment samples with sufficient mass remaining after DNA extraction and EPS analysis (tanks 2, 4, 6, 7) were analyzed for various metals according to EPA Method 200.7 by WVU’s Institute for Sustainability and Energy Research (WISER) analytical lab.^40^

### 2.7 Statistical Analyses

The Kruskal–Wallis Rank Sum Test was used to assess differences in α diversity, followed by posthoc pairwise Wilcoxon rank sum tests using RStudio (version 4.4.2).^41^ Nonmetric multidimensional scaling (NMDS) was applied to the classified OTUs at the species level for visualization of β diversity using code adapted from Torondel et al.^42^ by employing the *vegan* package^43^ in RStudio with Bray–Curtis dissimilarity. Analysis of similarities (ANOSIM) was conducted using *vegan* ^43^ with code adapted from Zorz.^44^ Mantel tests were performed using *vegan* ^43^ to assess correlations between bacterial community composition based on Bray–Curtis dissimilarities of species-level OTU data and distribution system characteristics and water quality using Euclidean dissimilarity. Biomarkers for sample groups were determined using linear discriminant analysis effect size (LEfSe) analysis via the *lefser* function^45^ in RStudio (refer to Text S6 for detailed methods). Figures were created by using Microsoft Excel and the *ggplot2* package on RStudio.

## 3 Results

### 3.1 Bacterial Community Composition and Diversity

#### 3.1.1 Bacterial Community Composition—Water and Sediment

All water samples were negative for total coliforms and *E. coli*, meeting regulatory compliance requirements. The sequencing results indicated a rich bacterial community throughout the sampled tanks. Thirty different bacterial phyla were detected across all samples, with 27 phyla found in water samples (distinct phyla: Hydrogenedentes, Caldiserica, Tenericutes, and Cloacimonetes) and 26 in sediment samples (distinct phyla: Omnitrophicaeota, FCPU426, and Zixibacteria) (Figure 2). Proteobacteria were the most abundant across all samples (average relative abundance 76 ± 25.4%), followed by Firmicutes (11.7 ± 14.8%) and Bacteroidetes (7.1 ± 12.7%). The Proteobacteria phylum was dominated by Alphaproteobacteria (53.5 ± 43.1%), Gammaproteobacteria (22.0 ± 25.2%), and Deltaproteobacteria (0.5 ± 1.0%). Alphaproteobacteria dominated most water samples (65.6 ± 42.5%), while Gammaproteobacteria were more common in sediment samples (44.4 ± 22.2%). Tanks 1 and 2 exhibited greater variation at the phylum and class levels compared to water samples from other tanks.

**Figure 2:**
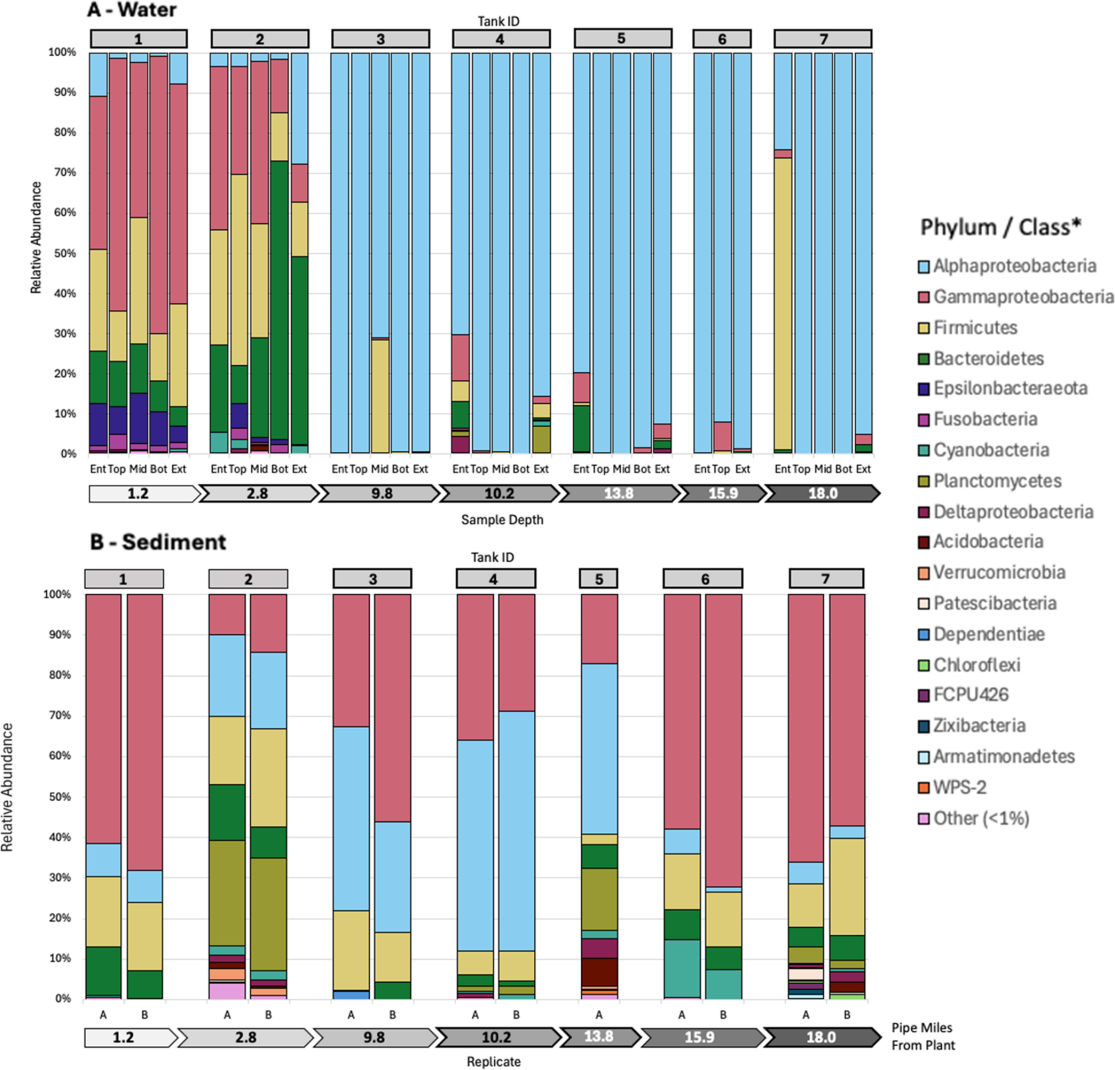
Relative abundance of taxa within water (A) and sediment (B) samples, grouped by tank ID (i.e., site) and sample depth within each tank. “Other” represents any phylum or class in every sample with less than 0.5% relative abundance. *Proteobacteria are split into a class, while all other taxa are at the phylum level. Abbreviations: Ent = entering, Mid = middle, Bot = bottom, and Ext = exiting.

#### 3.1.2 Bacterial Diversity

Differences in α diversity, which reflect species richness and evenness, were significant between tank sites (Kruskal–Wallis: χ^2^ = 22.106, *p* = 0.001158) and between sample types (i.e., water vs sediment; χ^2^ = 8.6417, *p* = 0.003286; Figure S1). However, α diversity differences based on sample depth were not statistically significant (χ^2^ = 2.1039, *p* = 0.7167; Figure S2). A posthoc Wilcoxon test was used to further assess any significant differences among sample depths within the tank. This analysis concluded that three pairs of sample depths/locations, bottom versus exiting (*V* = 0, *p* = 0.03125), entering versus exiting (*V* = 27, *p* = 0.03125), and exiting versus sediment (*V* = 2, *p* = 0.04688), exhibited significant differences in α diversity. The most pronounced significant difference between bacterial communities based on β diversity (i.e., community composition and abundance) was observed when grouped by tank site for both water (ANOSIM; *R* = 0.5630, *p* = 0.0001; Figure 3) and sediment (*R* = 0.7870, *p* = 0.0002; Figure S3). There was a significant but weaker difference between the bacterial communities in water compared with sediment samples across all tank sites (*R* = 0.2166; *p* = 0.0043; Figure S4). Sample depth did not show significant differences within the bacterial communities (*R* = −0.0963, *p* = 0.9981; Figure S5).

**Figure 3:**
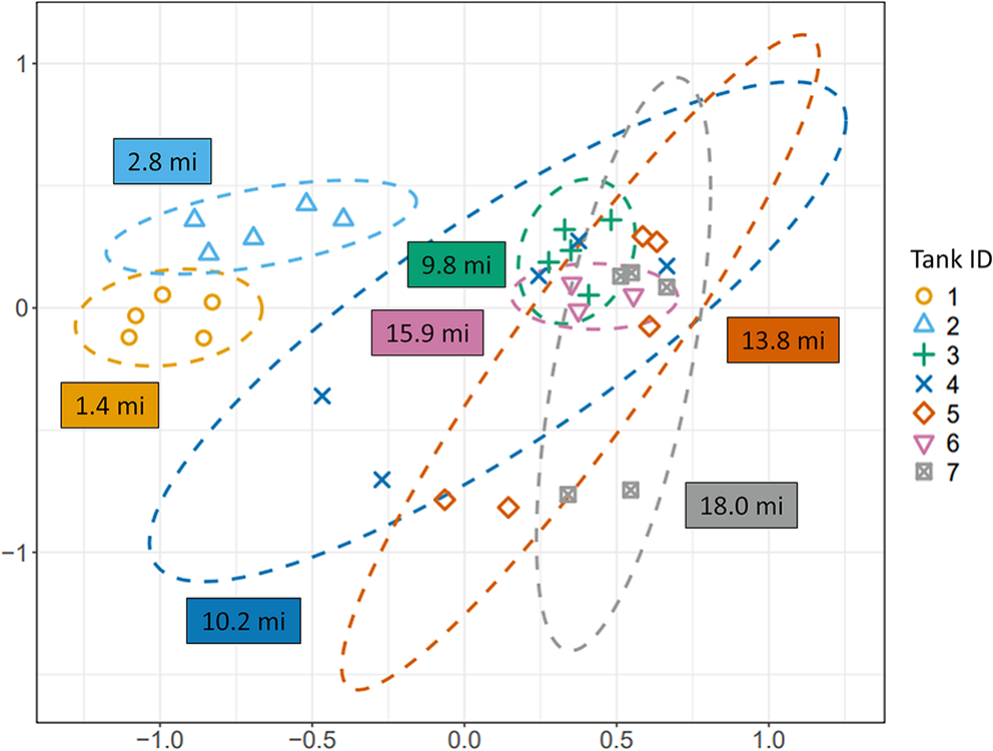
Nonmetric multidimensional scaling plot based on the β diversity of water samples, grouped by tank site. Boxes indicate pipe miles from the drinking water treatment plant.

### 3.2 Drivers of Bacterial Community Composition

#### 3.2.1 16S rRNA Gene Quantity

Water samples contained approximately 1–3 log_10_ 16S rRNA gene copies per milliliter, while sediment samples contained 5–8 log_10_ gene copies per gram. In water samples, 16S rRNA gene copy quantities generally increased with increasing distance from the DWTP (Figure 4). A similar trend was not observed in sediment samples. However, a notable spike in 16S rRNA gene copy abundance per gram of sediment was detected at tank 5, despite a recorded tank cleaning in 2020.

**Figure 4:**
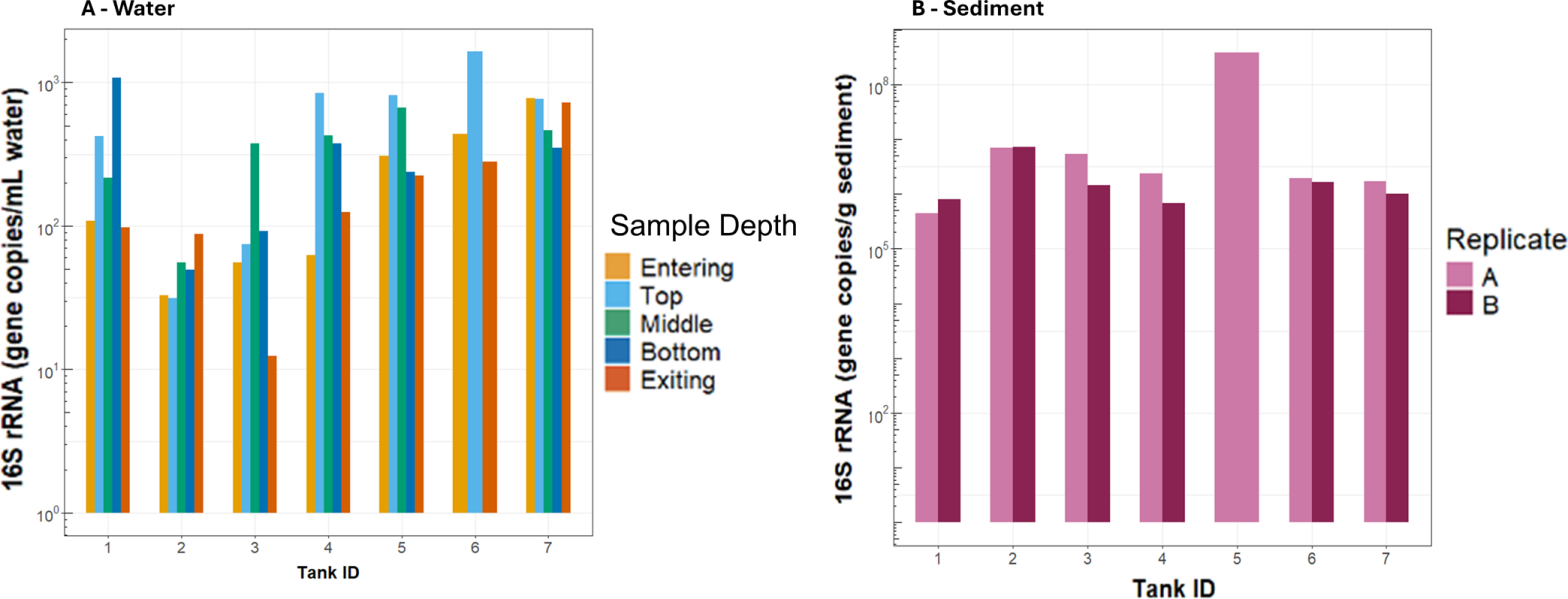
Normalized 16S rRNA gene copy quantities in water (A) and sediment samples (B).

#### 3.2.2 Distribution System Characteristics and Water Quality Impact on Bacterial Communities

Significant correlations were noted between bacterial communities and several key water quality parameters, including pH (Mantel; *r* = 0.263, *p* = 0.0054), conductivity (*r* = 0.213, *p* = 0.002), TDS (*r* = 0.208, *p* = 0.0021), total chlorine (*r* = 0.473, *p* = 0.0001), and TOC (*r* = 0.194, *p* = 0.0139), and distribution system characteristics, including pipe miles from the DWTP (*r* = 0.661, *p* = 0.0001) and tank turnover rate (*r* = 0.267, *p* = 0.0027) (Table S4). All physicochemical water quality results are given in Tables S5–S15.

#### 3.2.3 Impact of Chlorine

This study explored the relationship between chlorine residual and several key parameters, including 16S rRNA gene abundance, α diversity, and TTHMs. Chlorine residuals could be detected in all collected water samples (0.07–1.22 mg/L). A comparison of bacterial 16S rRNA gene copy abundance with chlorine residual showed that bacterial abundance generally increased as total chlorine levels decreased (Spearman’s rank correlation: ρ = −0.692, *p* = 0.000008; Figure S6). α diversity was correlated with chlorine residual, with tank sites closer to the DWTP exhibiting both higher α diversity and chlorine residual levels (ρ = 0.553, *p* = 0.0008; Figure S7). TTHMs were negatively correlated with chlorine residual concentration (ρ = −0.723, *p* = 0.000002; Figure S8), with the highest TTHM concentrations detected at tank 7, the tank furthest from the DWTP. All TTHM levels detected in this study were well below the regulatory limit of 0.080 mg/L.

#### 3.2.4 Sediment Composition

Elemental analysis of sediment revealed that iron, aluminum, and calcium were the most abundant of the measured metals across all samples (Figure S9). Specifically, tanks 2 and 4 were dominated by iron, with concentrations of 107,635 and 20,843 ppm, respectively. Calcium was most abundant in the sediment from tank 6 (21,480 ppm), while aluminum was highest in the sediment from tank 7 (5029 ppm). The average protein concentration was 0.00458 ± 0.00158 mg/g, while the average polysaccharide concentration was 0.01610 ± 0.00528 mg/g. The total EPS content across sediment samples averaged 0.02067 ± 0.00652 mg/g and remained relatively consistent across tank sites (Table S16).

#### 3.2.5 Detection of Pathogen-Containing Genera

*Stenotrophomonas*, *Acinetobacter*, and *Pseudomonas* were detected via 16S rRNA gene amplicon sequencing at every sample depth across all sites at least once (Figure 5). Other relevant pathogen-containing genera that were detected less frequently included *Aeromonas*, *Sphingomonas*, *Legionella*, *Burkholderia-Caballeronia-Paraburkholderia*, *Achromobacter*, and *Mycobacterium.* An in-depth exploration of biomarkers was conducted, and the results are given in Texts S7 and S8 and Figures S10–S12.

**Figure 5:**
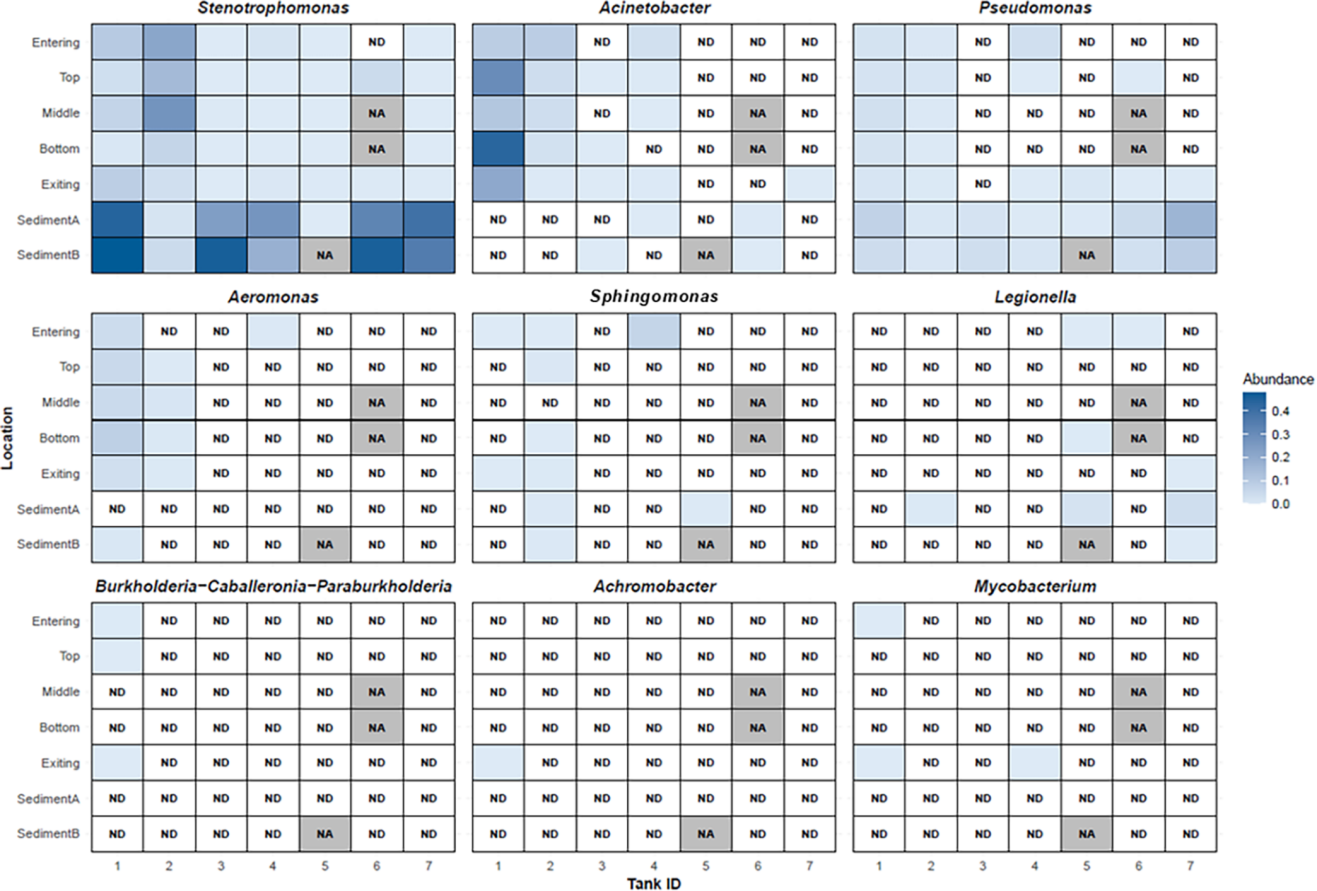
Relative abundance of key pathogen-containing genera by sample depth/location within each tank.

## 4 Discussion

### 4.1 Bacterial Community Composition of Water and Sediment

Proteobacteria was the dominant bacterial phylum across both tank water and sediment in the DWDS examined in this study, which aligns with findings from other studies exploring chlorinated DWDS microbial composition.^7,11,28,46,47^ Among Proteobacteria, Alphaproteobacteria is typically the dominant class in DWDSs^7,48,49^ and was similarly the most abundant in tank bulk water samples in this study. Alphaproteobacteria often thrive in DWDSs due to their adaptability to oligotrophic conditions.^50^ Gammaproteobacteria, Firmicutes, and Bacteroidetes were also dominant in bulk water samples and have been frequently reported in other DWDS studies.^7,11,28,51^

Sediment samples were dominated by Gammaproteobacteria and exhibited more consistent bacterial community composition across tanks, with little variation associated with distance from the DWTP compared to water samples. Gammaproteobacteria are commonly associated with biofilms and loose deposits, which likely explains their high abundance in settled tank particles.^19,48,52^ Notably, Ferrebee et al. found that bulk water samples were also dominated by Gammaproteobacteria in a chlorinated DWDS with relatively high biomass and loose deposits.^28^ This is significant because Gammaproteobacteria represent a large and diverse class of Proteobacteria, including many drinking water-associated pathogens.^53^ Disturbance of tank sediment could therefore pose a potential risk of contaminating distribution system water, as tank maintenance activities have been associated with several previous contamination events: a 1993 *Salmonella typhimurium* outbreak in Gideon, Missouri,^54^ a 1993 *Campylobacter jejuni* outbreak in Minnesota,^55^ and widespread coliform contamination in 1995 in Maine^3^ and in 2000 in Massachusetts.^3^ El-Chakhtoura et al. found that DWDS sediment communities largely determine water microbial quality,^56^ but our study indicated a lack of congruence between sediment and water bacterial community composition across tanks. This aligns with prior research indicating that bacterial composition typically differs between bulk water samples and loose sediment deposits.^19,57^

### 4.2 Spatial Variations Strongly Impact Bacterial Community Composition

#### 4.2.1 Community Shifts Associated with Tank Site Variability

Statistically significant differences in α diversity were observed between tank sites and between water and sediment samples, while differences between sample depths were not significant. This study demonstrated that the distance of tanks from the DWTP influenced the bacterial composition in bulk water samples, with greater α diversity observed in tanks closer to the plant. Greater α diversity was found in tanks with higher levels of chlorine residual, which counters the findings of various previous studies of distribution system water samples,^49,58–60^ although it is worth noting that few previous studies have examined α diversity in tanks. This discrepancy may be attributable to differing hydraulic and ecological conditions in storage tanks versus distribution systems. Sediment resuspension, flow dynamics, and chlorine tolerance could all impact α diversity levels in tanks. Further research is necessary to identify differences in the bacterial community in storage tanks compared to that of the entire DWDS to identify trends across system tanks. Spatial variation between tank sites had the strongest influence on bacterial diversity, suggesting that site-specific factors such as tank size, water age, and chlorine residual play a major role in shaping bacterial communities.^11,61^ While few studies have examined spatial variations when comparing DWDS tanks, several previous studies have found spatial variability to be a strong driver of bacterial communities in DWDSs.^11,20,61,62^ Statistically significant differences in the β diversity were noted between bacterial communities at different tank sites for both water and sediment samples. A slight increase in 16S rRNA gene copy abundance was observed in water samples at greater distances from the DWTP, which could indicate bacterial regrowth associated with chlorine residual decay. However, this was not the case for sediment samples, suggesting that sediments remain a microbially active environment regardless of the overarching spatial trends that drive bulk water quality.

#### 4.2.2 Community Shifts between Water and Sediment

In this study, β diversity metrics indicated significant differences between water and sediment, with greater α diversity measured in sediment than water. α diversity within water samples was relatively low (Shannon diversity index: 0.5–5.7), consistent with other studies of DWDSs.^46,47^

The bulk water from storage tanks in this DWDS exhibited lower abundances of 16S rRNA genes compared to levels reported in other DWDS studies.^7,63,64^ Quantities of the 16S rRNA gene were up to five times higher in sediment than in bulk water, reinforcing the idea that sediments may act as reservoirs for microbes.^65,66^ Loose deposits and particles tend to settle in low-flow environments, such as tanks, and provide a stable environment with sufficient nutrients from settled organic matter to support bacterial growth.^10,15,67^ In addition, residual disinfectants are unable to penetrate beyond the surface layers of sediment.^68^ These findings align with a previous study conducted by Liu et al. that found 98% of total bacteria in a DWDS were associated with loose deposits and pipe wall biofilm and approximately 2% were found in bulk water.^19^

#### 4.2.3 Community Shifts Associated with Sample Depth within Tanks

While limited research has explored bacterial variations within tanks, potential stratification and stagnation could lead to microbiome differences at different sampling locations.^61^ In this study, differences in α diversity, β diversity, and 16S rRNA gene abundance based on sample depth were not significant, suggesting that engineered flow dynamics may reduce stratification and support relatively uniform bacterial communities throughout the studied tanks.^4^

Although the sample depth within the tanks was not a significant factor overall, it is important to acknowledge the limitations of the sampling strategy. Increasing replication and recurrent sampling from specific depths within individual tanks would enhance insight into bacterial variation within tanks. The nature of the samples collected in this study promotes the comparison of tanks across the DWDS, rather than within them. While variation within the tanks was minimal, a significant difference in α diversity was noted between the tank influent and effluent water, highlighting the potential impact of tank storage on system water quality. Changes in bacterial diversity throughout tank residence may result from several factors, including microbial competition, nutrient availability, and particle settling. For example, sensitive taxa may die off during storage due to competition with more resilient taxa or through elimination by amoebal interactions, allowing only the more resilient species to persist.^69^ Similarly, the settling of particles during tank storage could reduce microbial loading in bulk water, enhancing it in sediments.^66,70^ Further research is needed to assess the influence of these processes and their effect on spatial variations in bacterial communities within storage tanks.

### 4.3 Distribution System Characteristics and Water Quality Impact on Bacterial Communities

Pipe miles from the DWTP and total chlorine had strong, positive correlations with the Bray–Curtis distance matrix of the bacterial community, while pH, conductivity, TDS, TOC, and tank turnover rate had positive but weaker relationships. The strong influence of total chlorine supports previous findings reporting that disinfectant decay shapes abundance and composition of bacterial communities as water travels through DWDSs.^11,28,51^ The positive correlation with tank turnover rate suggests that retention time also plays a role in bacterial community composition, as low turnover in storage tanks is linked to poor mixing, increased water age, and therefore potential water quality degradation.^4^ Turnover rates in the tanks sampled in this study were consistently high, which may explain the lack of variation between sample depths, likely attributed to reduced water age and minimal stratification. However, it is important to highlight that the turnover rate values were provided by utility personnel, who acknowledged that these rates were estimates based on snapshots in time, with a lack of temporally robust and well-validated supporting modeling data.

### 4.4 Sediment

Distribution-specific characteristics such as source water, treatment process, and DWDS materials all influence the composition of tank sediment.^14^ Of the elements measured in available sediment samples, iron was the most abundant on average, followed by aluminum and calcium. The dominance of iron suggests the presence of corrosion byproducts, which may be from pipe material, tank hinges, or interior corrosion of storage tank infrastructure.^3,15^ Aluminum may be coming from the aluminum sulfate that was fed at the plant as a coagulant, while calcium levels are likely attributable to calcium carbonate that forms from naturally occurring minerals paired with the lime treatment used for corrosion control. There was notable variation in elemental concentrations across tanks, indicating that sediment throughout the DWDS differs in makeup, likely contributing to differences in the sediment bacterial community based on micronutrient availability and metal toxicity.^71^ This aligns with findings from a previous study by Lytle et al., who reported differences in elemental concentrations from multiple tanks in the same DWDS.^14^ Consistent detection of both EPS and 16S rRNA genes in sediment samples demonstrates that the tank sediment collected in this study is rich in biomass and associated bacterial activity.

### 4.5 Detection of Pathogen-Associated Genera

Nine genera containing potentially pathogenic species were detected across the samples. *Stenotrophomonas* was the most frequently detected. Furthermore, the highest relative abundance values for the detected OPs were measured for *Stenotrophomonas*, particularly in sediment, suggesting that sediment may provide a favorable environment for pathogen survival and growth in DWDSs.

Other commonly detected pathogen-associated genera include *Acinetobacter* and *Pseudomonas*. Less frequently observed but still present were *Aeromonas*, *Sphingomonas*, *Legionella*, *Burkholderia-Caballeronia-Paraburkholderia*, *Achromobacter*, and *Mycobacterium*. Other studies have detected *Legionella pneumophila* and *P. aeruginosa* in tank sediments and DWDS.^9,11,72^ Exposures to these OPs pose health risks, as they are leading sources of waterborne disease outbreaks.^73^ The occurrence of *L. pneumophila* in DWDS can cause legionellosis and Legionnaires’ disease, although it is uncommon in well-maintained systems for detection levels to be associated with public health risks.^16,74^ However, storage tank conditions can influence microbial risk; LeChevallier linked various tank attributes such as water stagnation, increased water temperatures, sediment accumulation, and inadequate mixing to water quality degradation, promoting *Legionella* detection.^75^ Another study conducted by Cohn et al. found legionellosis cases were occurring near storage tanks.^74^ Previous studies have identified OPs from other noted genera in DWDSs, including *Acinetobacter baumannii*,^64^ *Stenotrophomonas maltophilia*,^76^ *P. aeruginosa*,^7,64,73^ and *Aeromonas hydrophila*.^64^ There is limited knowledge of the public health risks corresponding to the presence of OPs in drinking water, creating the need for further monitoring and research of these organisms, especially in tanks.^17^

The highest occurrence of these OP-associated genera was found in tanks closest to the DWTP (tanks 1 and 2). This finding differs from previous studies that link the growth of OPs to increased water age and decreased disinfectant residual.^17^ The detection of potentially pathogenic genera in tanks with higher disinfectant levels and shorter distribution times may point to the presence of chlorine-resistant taxa or other system specific conditions that support the survival of these genera.^77,78^ It is essential to note that the detection of genera containing pathogenic species can often occur in DWDSs without an associated human disease burden, and this study did not quantify pathogenic species. Sequences do not always represent pathogenic species and in turn do not necessarily indicate a risk to human health.^76^

## 5 Conclusions

This study investigated the bacterial community composition of water and sediment from storage tanks in a chlorinated DWDS, revealing clear distinctions between sampled water and sediment. Sediment provided a favorable environment for potentially pathogenic genera such as *Stenotrophomonas*, *Pseudomonas*, and *Acinetobacter*, which were detected across nearly all sites. Recent tank cleanings did not correspond to reduced bacterial abundance and, in one case, were associated with a spike in sediment bacterial load. Although genera containing potentially pathogenic species were detected, the presence of pathogenic strains was not confirmed and total coliforms and *E. coli* remained undetectable in all water samples, indicating compliance with regulatory standards.

Overall, these findings highlight the influence of spatial variation, tank-specific conditions, and sediment accumulation on the bacterial communities within DWDS storage tanks. These findings also highlight the need for a continued exploration of storage tank sediments and variations within the tank to inform maintenance strategies, including the frequency and occurrence of tank cleanings, as DWDS storage tanks play an important role in the water quality delivered to customers.

## Supplementary Material



## Notes

Additional methods including sample collection (Text S1); physicochemical analysis methods (Text S2); quantification of 16S rRNA genes (Text S3) including component volumes (Table S1), thermocycler conditions (Table S2), and qPCR and PCR primers (Table S3); 16S rRNA gene amplicon sequencing (Text S4); characterization of sediment (Text S5); determination of biomarkers (Text S6); biomarker results (Text S7); discussion of pathogenic biomarkers (Text S8); α diversity grouped by sample type and sample depth (Figures S1 and S2); NMDS of sediment samples grouped by site (Figure S3); NMDS grouped by sample type (Figure S4); NMDS of water samples by sample depth (Figure S5); chlorine residual vs bacterial abundance (Figure S6); chlorine residual vs α diversity (Figure S7); chlorine residual vs TTHMs (Figure S8); elemental concentration of tank sediment (Figure S9); LDA effect size for genera between tank sites for water samples and sediment samples (Figures S10 and S11); and LDA effect size for genera between water and sediment (Figure S12); Mantel test results (Table S4); physicochemical water quality parameters (Tables S5–S15); protein, polysaccharide, and total EPS content (Table S16); *R* ^2^ and efficiency values (Table S17) (PDF)
