# Supplementary material for: Divergent Bacterial Communities in Water and Sediment of Chlorinated Drinking Water Storage Tanks
Source: ACS ES T Water. 2025 Dec 11;6(1):217–27. doi: 10.1021/acsestwater.5c00928 (PMC12797242; doi:10.1021/acsestwater.5c00928)
Supplement: Supplementary file 1 [file ew5c00928_si_001.pdf]

Supplementary Materials

Divergent Bacterial Communities in Water and Sediment of Chlorinated Drinking Water Storage Tanks

Eva Bridges,[1] Sienna Bircher,[1] Kara Cunningham,[1] Vinila Vasam,[1] John Hando,[2,3] Emily Garner[1]*

[1]Wadsworth Department of Civil & Environmental Engineering, West Virginia University, Morgantown, West Virginia, 26506, United States

[2]Department of Industrial and Management Systems Engineering, West Virginia University, Morgantown, West Virginia, 26506, United States

[3]School of Natural Resources and the Environment, West Virginia University, Morgantown, West Virginia, 26506, United States

*Corresponding Author

E-mail: emily.garner@mail.wvu.edu

Content

Text S1: Sample collection

Text S2: Physicochemical analysis methods

Text S3: Quantification of 16S rRNA genes

Table S1: Component volumes per reaction used for qPCR amplification.

Table S2: Thermocycler conditions used for qPCR amplification.

Table S3: qPCR and PCR primers.

Text S4: 16S rRNA gene amplicon sequencing

Text S5: Characterization of sediment

Text S6: Determination of biomarkers

Text S7: Biomarkers

Text S8: Pathogenic biomarkers

Figure S1: Alpha diversity of samples, grouped by sample type (i.e., water vs sediment) and tank ID (i.e., site).

Figure S2: Alpha diversity of samples, grouped by sample depth within the tank and tank ID (i.e., site).

Figure S3: NMDS of sediment samples, grouped by tank site. Boxes indicate pipe miles from the drinking water treatment plant.

Figure S4: NMDS of all samples, grouped by sample type (i.e., water vs sediment).

Figure S5: NMDS of water samples, grouped by sample depth within tank.

Figure S6: Chlorine residual vs bacterial abundance (16S rRNA gene copies / mL) grouped by tank ID (i.e., site), sample depth within the tank, and pipe miles from the DWTP.

Figure S7: Chlorine residual vs alpha diversity grouped by tank ID (i.e., site), sample depth within the tank, and pipe miles from the DWTP.

Figure S8: Chlorine residual vs TTHMs grouped by tank ID (i.e., site), sample depth within the tank, and pipe miles from the DWTP.

Figure S9: Elemental concentration of tank sediment.

Figure S10: LDA effect size for genera between tank sites for water samples. Results shown are LDA > 2.5 and $p < 0.05$. Biomarkers with unknown or uncultured genera were excluded from the figure. Proteobacteria were split into class and all other taxa were identified at the phylum level.

Figure S11: LDA effect size for genera between tank sites for sediment samples. Results shown are LDA > 2.5 and $p < 0.05$. Biomarkers with unknown or uncultured genera were excluded from the figure. Proteobacteria were split into class and all other taxa were identified at the phylum level.

Figure S12: LDA effect size for genera between water and sediment. Results shown are LDA > 2.5 and $p < 0.05$. Biomarkers with unknown or uncultured genera were excluded from the figure. Proteobacteria were split into class and all other taxa were identified at the phylum level.

Table S4: Mantel test results of correlations between microbial community and distribution system characteristics. N = 33 for all parameters. Asterisks denote significance: *** $p \leq 0.001$, ** $p \leq 0.01$, * $p \leq 0.05$.

Table S5: pH of each sample depth at each tank site.

Table S6: Temperature (◦C) of each sample depth at each tank site.

Table S7: Conductivity (μs/cm) of each sample depth at each tank site.

Table S8: Total dissolved solids (ppm) of each sample depth at each tank site.

Table S9: Total dissolved solids (ppm) of each sample depth at each tank site.

Table S10: Total chlorine concentrations (mg/L) of each sample depth at each tank site.

Table S11: Iron concentrations (mg/L) of each sample depth at each tank site.

Table S12: Nitrite concentrations (mg/L) of each sample depth at each tank site.

Table S13: Nitrate concentrations (mg/L as NO_3) of each sample depth at each tank site.

Table S14: TTHM concentrations (mg/L) of each sample depth at each tank site.

Table S15: Total organic carbon (mg/L) of each sample depth at each tank site.

Table S16: Protein, polysaccharide, and total EPS content.

Table S17: R^2 and efficiency values.

Methods

Text S1: Sample collection

Bulk water collection

Tank water at three depths, tank influent and effluent water, and sediment samples were collected from each tank site. The sample depth within the tank depended on the selected tank size and sampling day operating range. Sterilized bacon bombs (George Taylor Brass Bronze Works) were attached to two pieces of braided, polyester rope that were used for lowering the device in order to minimize rotation of the sampling device. An attached string was also included on the devices to open and close the plunger at the desired depth. Through pulling the attached string to activate the device's plunger, isolated grab samples of approximately 1 L were collected. Each bacon bomb was lowered to its designated sampling depth five times to retrieve a total of approximately 5 L of water.

Sediment collection

At sample locations where greater sediment accumulation was present, a dredge (LaMotte™) was used to collect sediment. A dredge is a sampling device that has a trigger that holds the sampler open while lowering and closes when it contacts a surface which allows for collection of solids without excessive liquid. Varying quantities of sediment were collected due to differences in deposition at each tank site.

Sample transport and storage

Up to 3,400 mL of water sample was filter-concentrated onto a 0.2 µm mixed cellulose ester filter (MilliporeSigma) for molecular analysis. Smaller volumes of water were filtered for some samples due to larger amounts of sediment present which resulted in clogging.

Text S2: Physicochemical analysis methods

Various key physicochemical water characteristics were analyzed for each collection point on site (Figure S1). Temperature, pH, and conductivity were measured using a Hach HQ2100 multimeter. Total and free chlorine concentrations were analyzed via DPD Total Chlorine and DPD Free Chlorine methods (Hach Method #8167 and 8021, respectively) and measured using a DR900 colorimeter (Hach, Loveland, CO). Total dissolved solids (TDS) were measured using a Thermo Scientific ELITEPCTS Pocket Meter. After arrival at the lab, nitrite, nitrate, and total iron concentrations were measured using a Hach DR900 colorimeter and USEPA Diazotization Method (Hach Method #8507), Cadmium Reduction Method (Hach Method #8039), and USEPA FerroVer Method (Hach Method #8008), respectively. A 2100P Turbidimeter by Hach was used to measure turbidity according to Standard Method 2540D for each sample and total organic carbon (TOC) was measured using a Shimadzu Organic Carbon Analyzer according to Standard Method 5310B.

The Colilert enzyme substrate coliform test (IDEXX Laboratories, Inc., Westbrook, ME) was performed using 100 mL of each water sample and incubated for 24 hours at 35 ± 0.5°C according to Standard Method #9223 to test for total coliforms and *E. coli.*[1]

Text S3: Quantification of 16S rRNA genes

Quantitative polymerase chain reaction (qPCR) was performed via a QuantStudio 3 real-time PCR system (Applied Biosystems, Thermo Fisher, USA).

The reactions were performed using 2.4 µL of molecular grade water, 0.8 µL of previously published forward primer 1369F and reverse primer 1492R[2] at a stock concentration of 10 µM, 5.0 µL of 2X PowerUP SYBR Mastermix (Life Technologies), and 1 µL extracted template DNA. The following thermocycler conditions were used: 50℃ for 2 minutes, 95℃ for 2 minutes,

then 40 cycles of 95°C for 5 seconds, 55°C for 30 seconds, and 72°C for 30 seconds, followed by a melt curve in which temperatures were increased from 65 to 95°C in 0.5°C increments.

Table S1: Component volumes per reaction used for qPCR amplification.

**Component**	**Volume (µL)**
Molecular grade water	2.4
Forward primer (1369F)	0.8
Reverse primer (1492R)	0.8
2X PowerUp SYBR Master Mix	5.0
Extracted template DNA	1.0
**Total volume**	10.0

Table S2: Thermocycler conditions used for qPCR amplification.

**Thermocycler Step**	**Temperature (°C)**	**Duration**	**Cycles**
Initial UNG activation	50	2 min	1
Initial denaturation	95	2 min	1
Denaturation	95	5 sec	40
Annealing	55	30 sec	40
Extension	72	30 sec	40
Melt curve	65-95 (0.5 steps)	Per step	--

Table S3: qPCR and PCR primers.

Purpose	Name	Primer Sequence	Length of PCR product (bp)	Source

qPCR	1369F	5’-CGGTGAATACGTTCYCGG -3’	124 bp	Suzuki et al., 2000
	1492R	5’- GGWTACCTTGTTACGACTT -3’		Suzuki et al., 2000
PCR (round 1)	8F	5’- AGAGTTTGATYMTGGCTCAG-3’	1,485 bp	Juretschko et al., 1998
	1492R	5’- GGWTACCTTGTTACGACTT -3’		Suzuki et al., 2000
PCR (round 2)	515F	5′-GTGYCAGCMGCCGCGGTAA	~450 bp (with barcodes/Illumina adapters)	Parada et al., 2016
	926R	5′-CCGYCAATTYMTTTRAGTTT		Quince et al., 2011

Text S4: 16S rRNA gene amplicon sequencing

Preparation of amplicon libraries was performed using a two-step nested-polymerase chain reaction (PCR) method, this approach was chosen because a single round of amplification did not yield sufficient DNA mass for all samples.

Round one of PCR had reactions composed of 10 µL Platinum™ Hot Start PCR Master Mix (2X), 0.5 µL of forward (8F) and reverse primers (1492R) at a stock concentration of 10 µM, 1 µL of template DNA extract, and 13 µL of molecular grade water for a final volume of 25 µL. Molecular grade water was used rather than template DNA extract for three negative controls per 24 samples processed. PCR amplification was performed in triplicate and the thermocycler

conditions were as follows: 3 minutes at 94°C for initial denaturation, 25 cycles of denaturation at 94°C for 45 seconds, annealing at 50°C for 60 seconds, and extension at 72°C for 90 seconds, with a final extension at 72°C for 10 minutes and a hold at 4°C after the run is complete. Triplicates of each sample at 25 µL were combined for a total volume of 75 µL and stored at -20°C until the second round was performed.

Round two reactions consisted of the same volumes as round one with the differences being the substitution of the respective primers and template DNA extract (i.e., PCR product from the first round of PCR). PCR amplification was performed using the same conditions as round one, except for the denaturation and annealing steps, which were increased to 35 cycles rather than 25. Triplicates of each sample at 25 µL were combined for a total volume of 75 µL and stored at -20°C until further use.

One percent agarose E-Gel™ was prepared with 14 µL of E-Gel™ sample loading buffer (1X) (Life Technologies) and 1 µL of PCR product for each sample to verify amplification. For each prepared gel, two wells per row were filled with GeneRuler 100 bp DNA Ladder (Life Technologies). Proper PCR amplification was confirmed through the observation of a bright band of approximately 450-500 bp. To ensure the absence of contamination, it was confirmed that the negative controls exhibited no detectable bands within the 450-500 bp range.

After quality filtering, 2,907 unique features were counted with lengths ranging from 362 – 480 base pairs. After removing blanks, samples had between 59,339 and 442,935 reads with an average of ~173,572 reads.

Text S5: Characterization of sediment

For extracellular polymeric substance (EPS) extraction, the extraction buffer was prepared by dissolving 0.328 g of $Na_3PO_4.12(H_2O)$, 0.552 g of $NaH_2PO_4.(H_2O)$, 0.526 g of NaCl, and 0.0746 g of KCl into 1 L of deionized (DI) water. The pH was adjusted to 7.0 using 1 M NaOH.

Before use, the CER was conditioned according to the manufacturer's instructions by thoroughly washing with the prepared extraction buffer (PBS) for one hour to remove any impurities.[3,4] In sterile 5 mL centrifuge tubes, 3 grams of sediment and 1.5 mL of PBS were combined, followed by 0.5 g of conditioned resin. The tubes were placed on an orbital shaker and incubated at 350 rpm for one hour at 15°C.[5] After extraction, crude EPS was centrifuged at 12,000 x g for 15 minutes to separate sediment solids from the EPS-rich supernatant. The supernatant was then filtered through a 0.2 μm syringe filter to remove any remaining particulates and stored at 4°C for further analysis.

Total protein content in the extracted EPS was measured using the bicinchoninic acid (BCA) method (Pierce BCA Protein Assay kit, Thermo Scientific).[6] The required reagent was prepared by mixing reagent A and B from the protein assay kit at a 50:1 ratio. In an acid-washed glass test tube, 2 mL of extracted EPS and 2 mL of prepared reagent were combined and thoroughly mixed. Bovine serum albumin (BSA) standards were prepared at concentrations of 0, 1, 2.5, 5, 7.5 and 10 mg/L in separate acid-washed glass test tubes. All samples and standards were gently mixed and incubated in a 60°C water bath for one hour to allow color development. After incubation, samples and standards were cooled to room temperature. A Thermo Scientific BioMate3 UV-Vis V spectrophotometer with a 1 mL glass cuvette was used to measure absorbance at 562 nm. The absorbance values of the BSA standards were used to generate a

linear standard curve, helping to determine the protein concentration in the EPS samples by interpolation.

Polysaccharide content of the extracted EPS was determined with the phenol-sulfuric acid method[7], in which carbohydrates react with sulfuric acid to produce a yellow-orange color that can be quantified spectrophotometrically. In acid washed glass test tubes, 0.5 mL of EPS extract was combined with 0.5 mL of 5% phenol (Sigma Aldrich) in water and 2.5 mL of concentrated sulfuric acid (98-99%). The tubes were sealed and gently inverted to mix. D-glucose standards were prepared at concentrations of 0, 1, 2.5, 5, 7.5, and 10 mg/L. To each prepared standard, 0.5 mL of phenol and 2.5 mL of sulfuric acid were added and mixed gently. All tubes were incubated in a 30°C water bath for 15 minutes to allow the color reaction to develop, then cooled to room temperature. Absorbance was measured at 490 nm using a 1 mL glass cuvette in the UV-Vis spectrophotometer. A second-degree polynomial standard curve was generated from glucose standards, enabling the calculation of the polysaccharide concentration in the EPS samples.

Text S6: Determination of biomarkers

Biomarkers were identified as features showing statistically significant differences in relative abundance between categorical groups. Biomarkers for sample groups were found using the *lefser* function in RStudio,[8] the package applies Kruskal-Wallis tests ($\alpha = 0.05$) followed by linear discriminant analysis (LDA) with threshold score of 2.5. Sample groupings analyzed independently included: water samples grouped by tank site, sediment samples grouped by tank site, and samples grouped by type (i.e., water vs sediment).

Results

Text S7: Biomarkers

The highest number of biomarkers were found for water samples grouped by tank site, closely followed by sediment samples grouped by tank site. Among water samples grouped by site, biomarkers mostly belonged to the Proteobacteria Phylum (43.9%), with Alphaproteobacteria (9.8%) and Gammaproteobacteria (34.1%) as prominent classes. Several biomarkers were genera that contain pathogenic species, including *Stenotrophomonas*, *Aeromonas*, and *Pseudomonas* at tank 1 while *Stenotrophomonas* and *Acinetobacter* were associated with tank 2. Notably, 92.7% of water sample biomarkers were concentrated at tank 1 and 2. Similarly, in sediment samples grouped by tank site, 50% of biomarkers were Proteobacteria, distributed among Alphaproteobacteria (14.3%), Gammaproteobacteria (32.1%), and Deltaproteobacteria (3.6%) with a more even distribution across tank sites. Genera containing pathogenic species were again detected, with *Pseudomonas* and *Legionella* emerging as biomarkers for tank 7 sediment.

When samples were grouped by type (i.e., water vs. sediment), there were fewer biomarkers detected in water samples compared to sediment samples (Figure S13). All biomarkers in water samples belonged to Gammaproteobacteria, while in sediment samples, half were from this class. *Acinetobacter* was identified as a biomarker for water and *Stenotrophomonas* and *Pseudomonas* were identified as biomarkers for sediment.

No significant features were detected when samples were classified by entering and exiting sample depths within the tank.

Discussion

Text S8: Pathogenic biomarkers

Three tank sites (tanks 1, 2, and 7) exhibited biomarkers of genera known to include pathogenic species. Water samples from the two tank sites closest to the treatment plant (1.4 miles and 2.8 miles away, respectively) and sediment samples from the furthest tank site (18 miles) contained potentially pathogenic biomarkers. In tank 1, these included *Stenotrophomonas, Aeromonas,* and *Pseudomonas* while tank 2 harbored *Stenotrophomonas* and *Acinetobacter.* The water-associated biomarkers identified may persist in tanks close to the treatment plant due to their known chlorine resistance, indicating that proximity to the treatment plant does not necessarily eliminate risk.[9,10]

Tank 7's sediment had two genera that contained pathogenic species as biomarkers including *Pseudomonas* and *Legionella*.

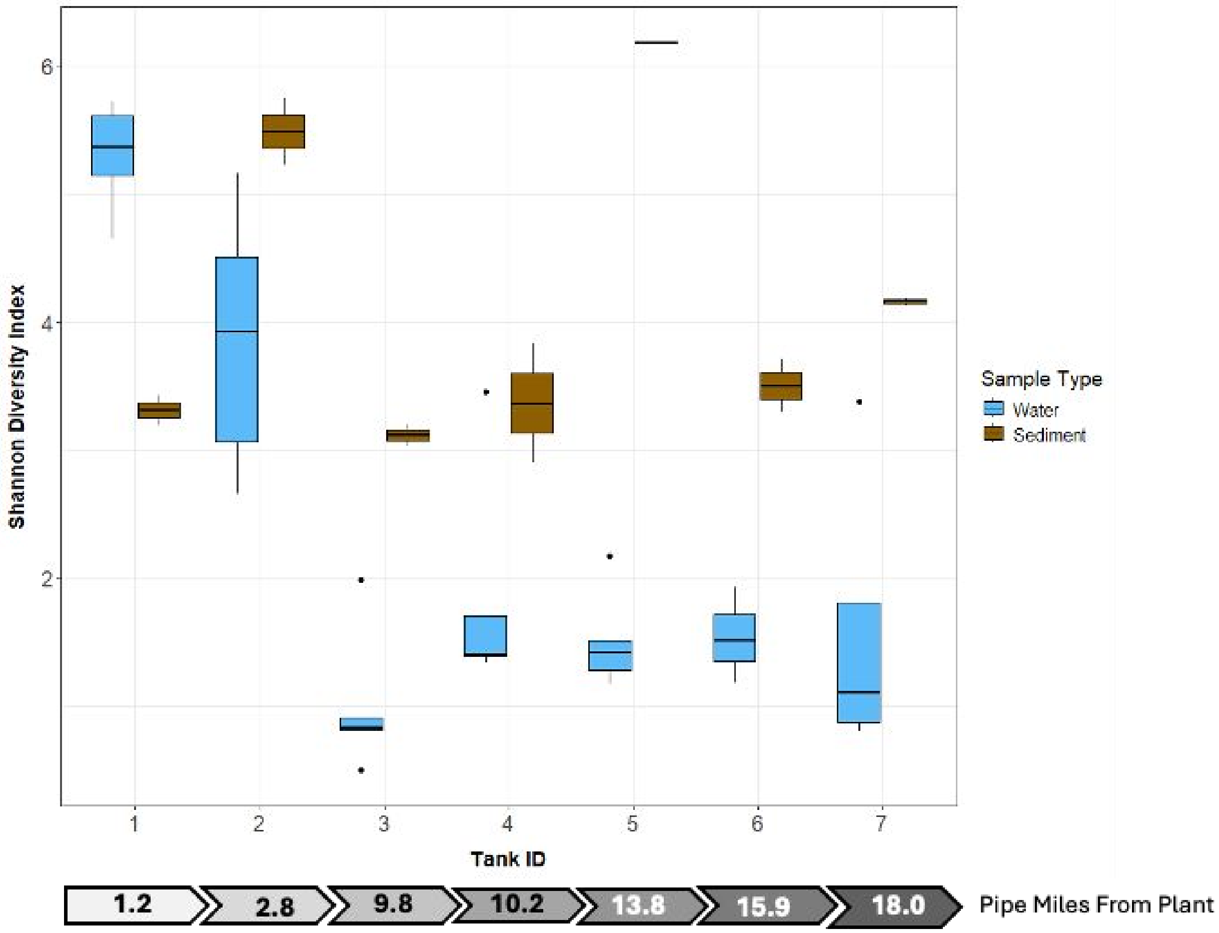

Figure S1: Alpha diversity of samples, grouped by sample type (i.e., water vs sediment) and tank ID (i.e., site).

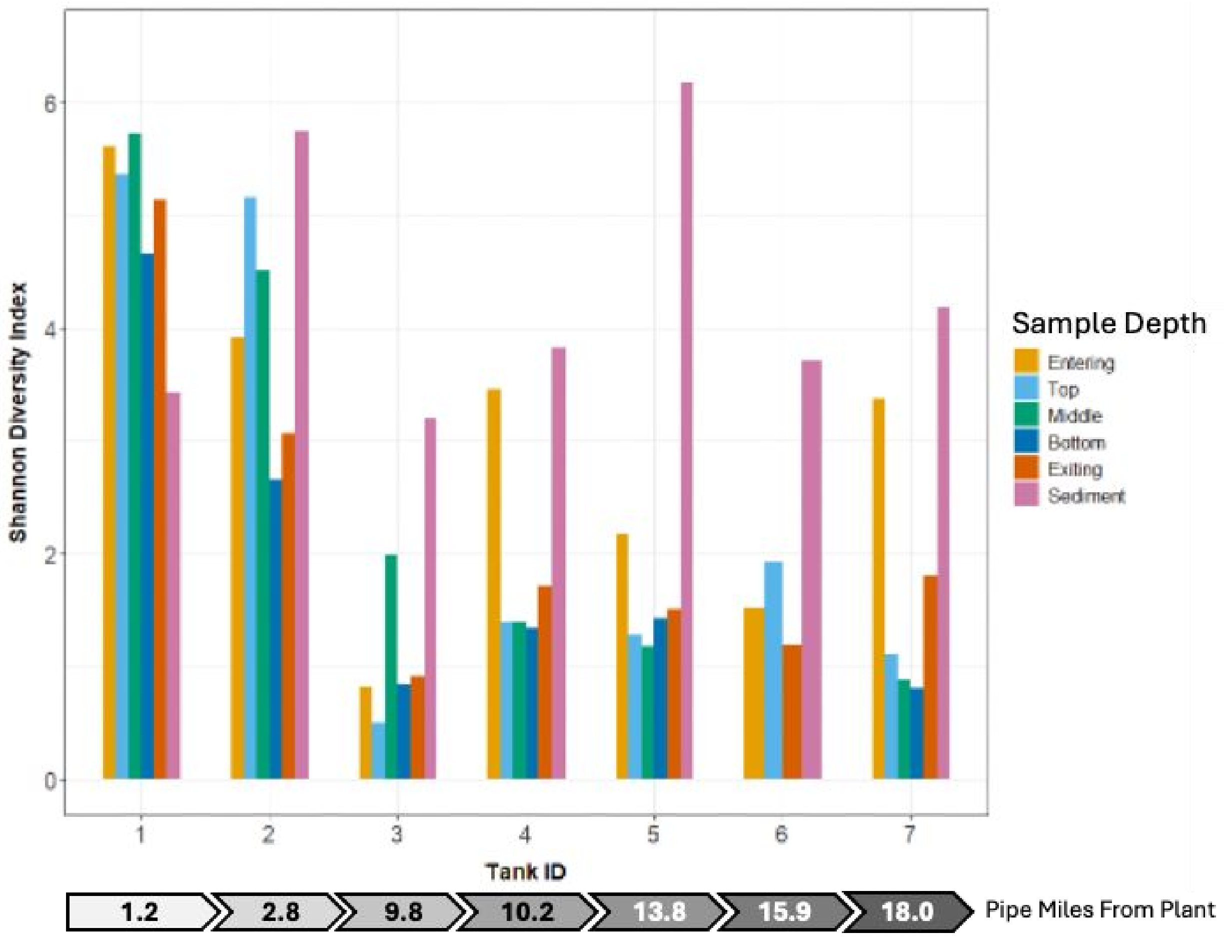

Figure S2: Alpha diversity of samples, grouped by sample depth within the tank and tank ID (i.e., site).

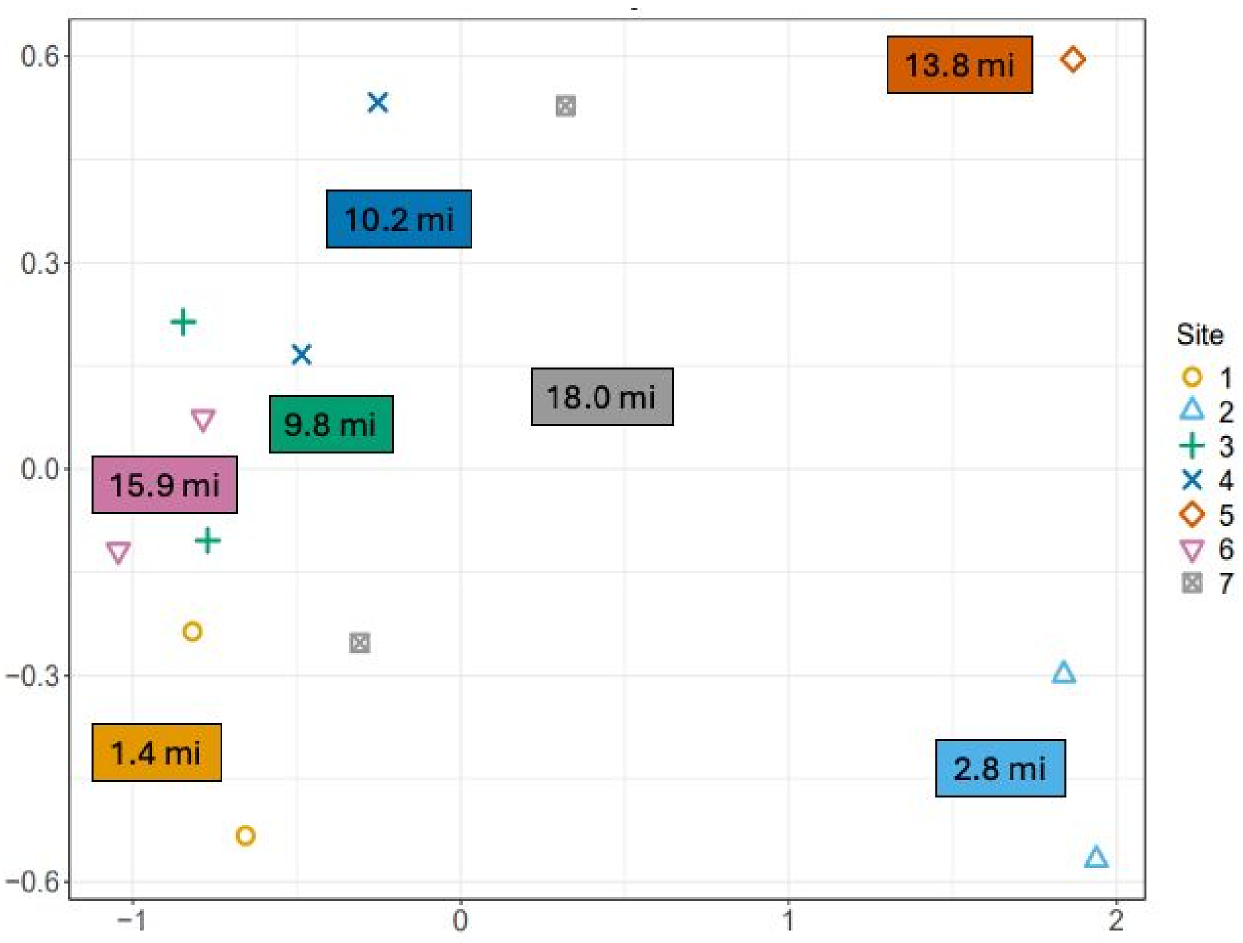

Figure S3: NMDS of sediment samples, grouped by tank site. Boxes indicate pipe miles from the drinking water treatment plant.

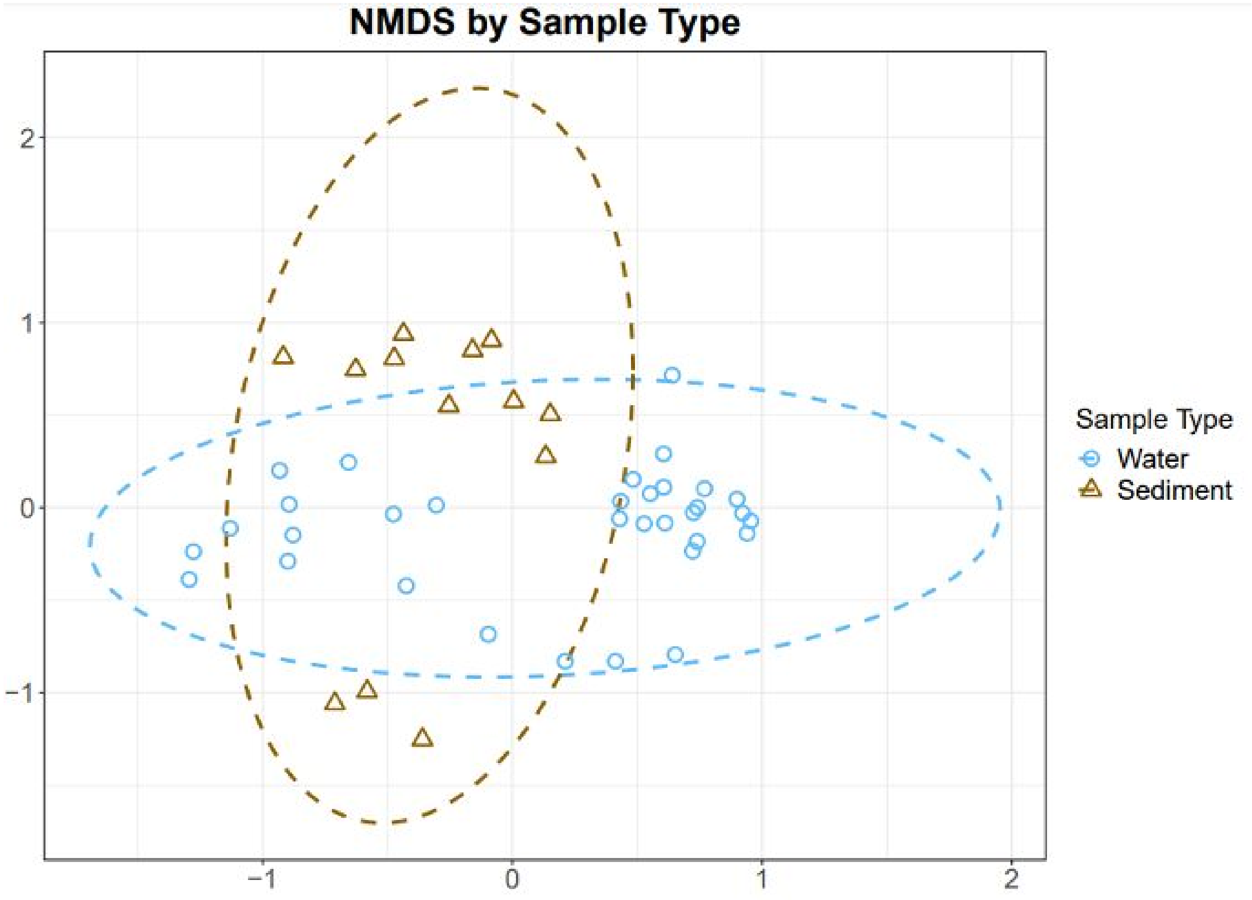

Figure S4: NMDS of all samples, grouped by sample type (i.e., water vs sediment).

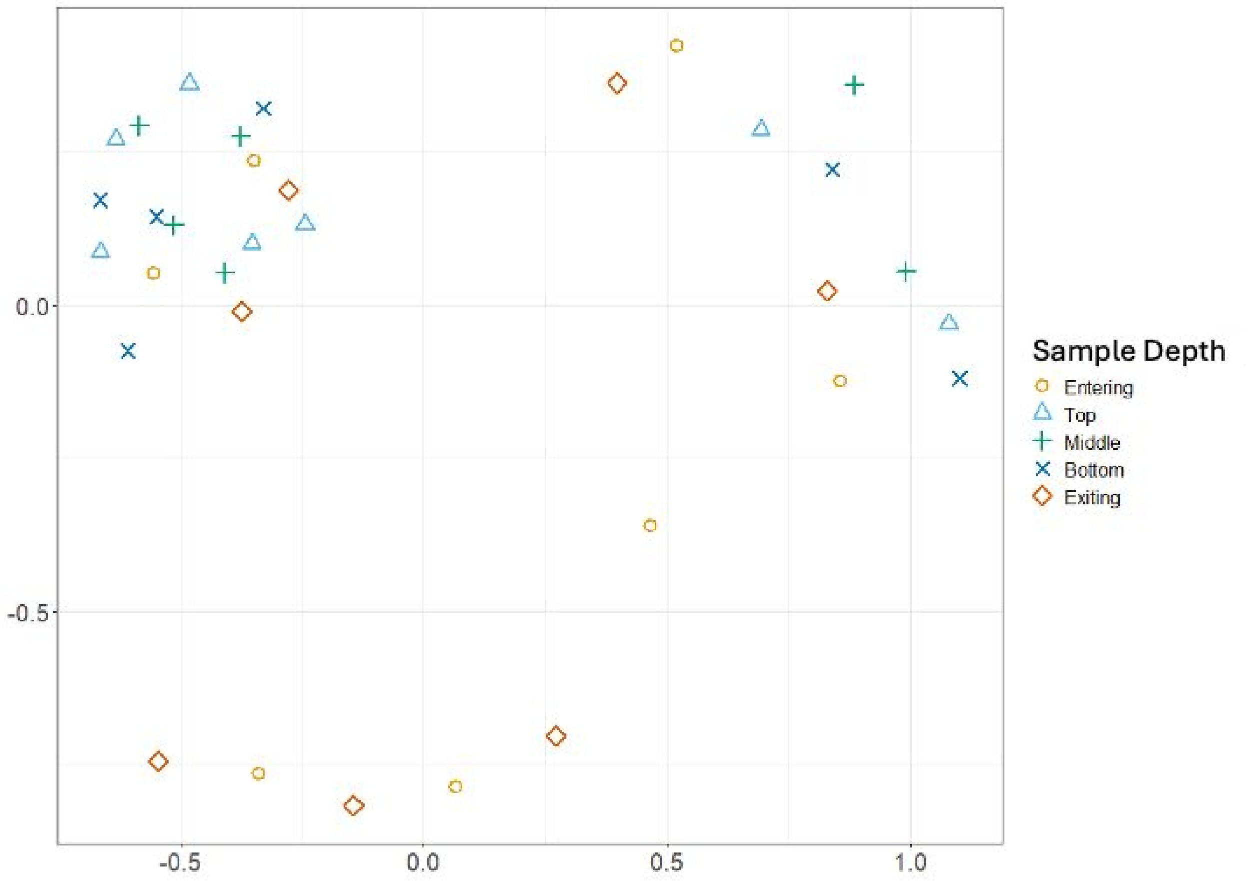

Figure S5: NMDS of water samples, grouped by sample depth within tank.

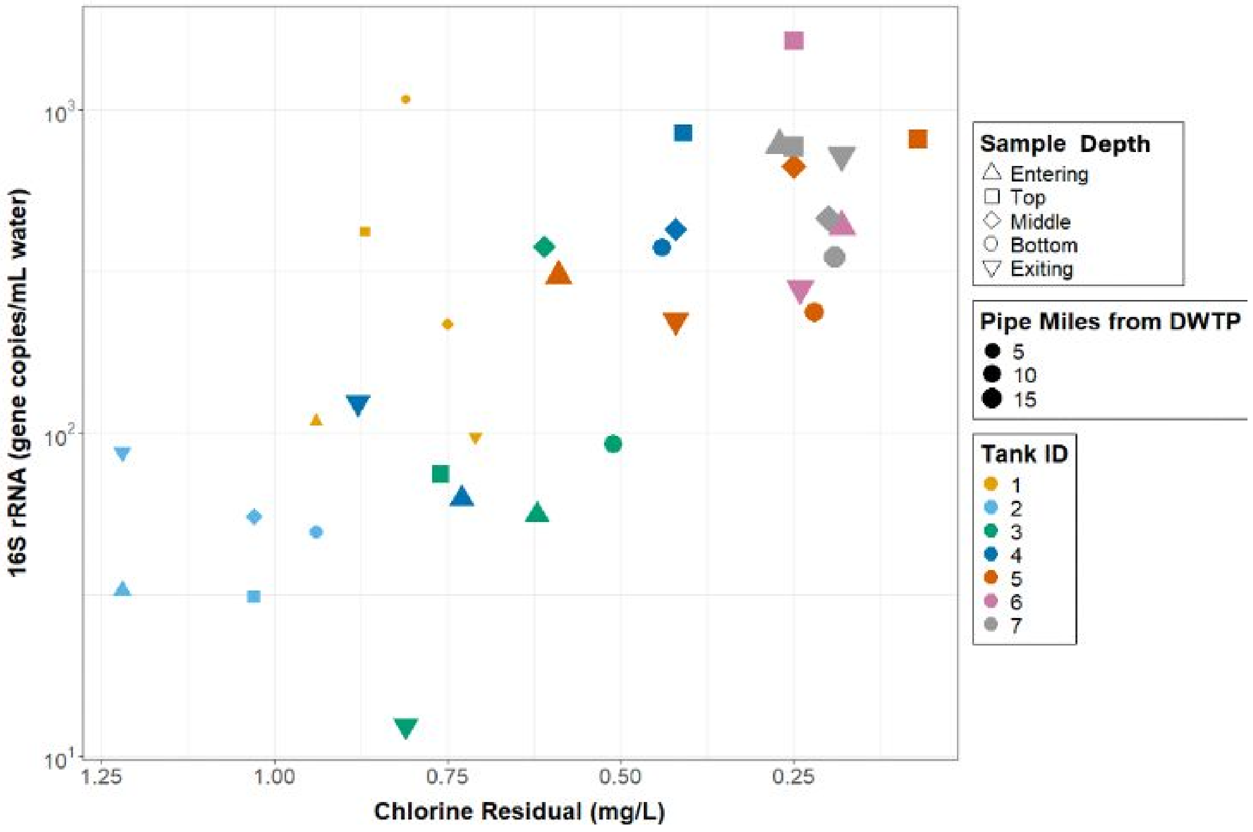

Figure S6: Chlorine residual vs bacterial abundance (16S rRNA gene copies / mL) grouped by tank ID (i.e., site), sample depth within the tank, and pipe miles from the DWTP.

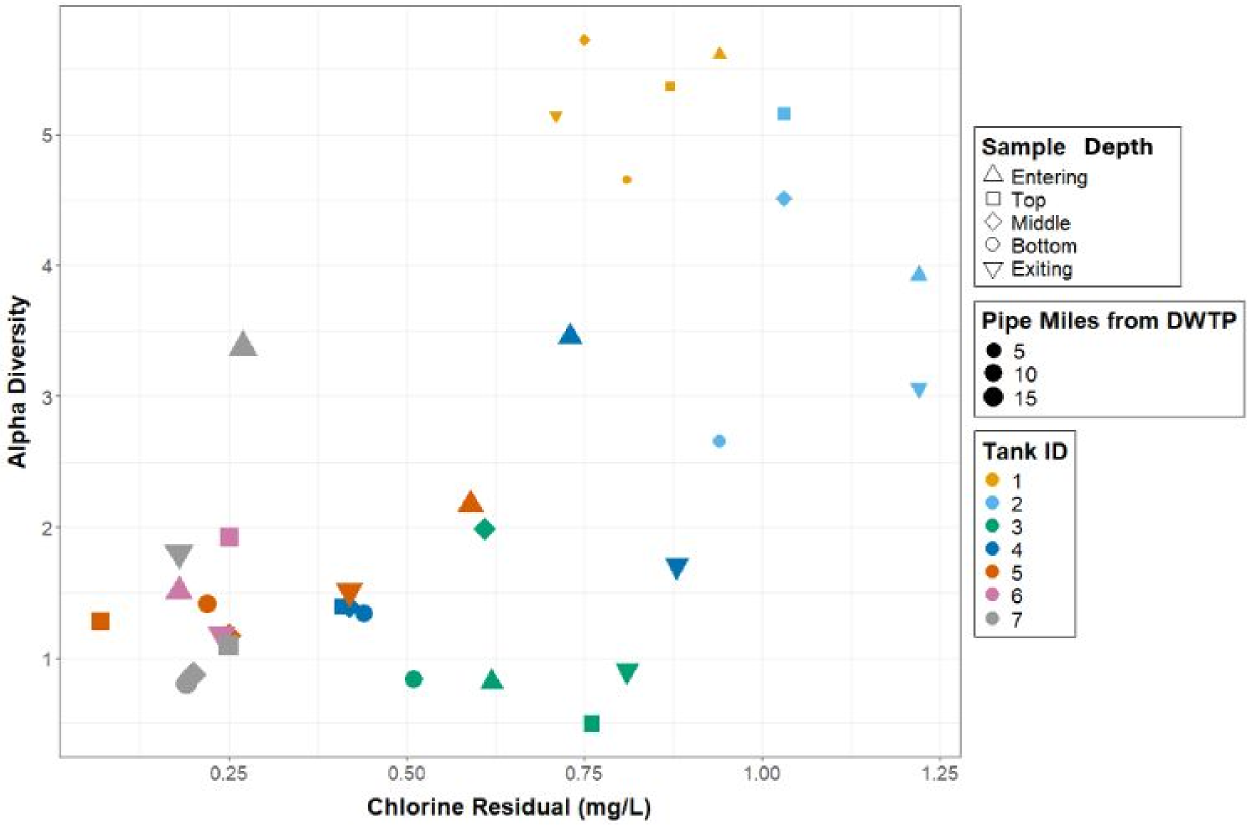

Figure S7: Chlorine residual vs alpha diversity grouped by tank ID (i.e., site), sample depth within the tank, and pipe miles from the DWTP.

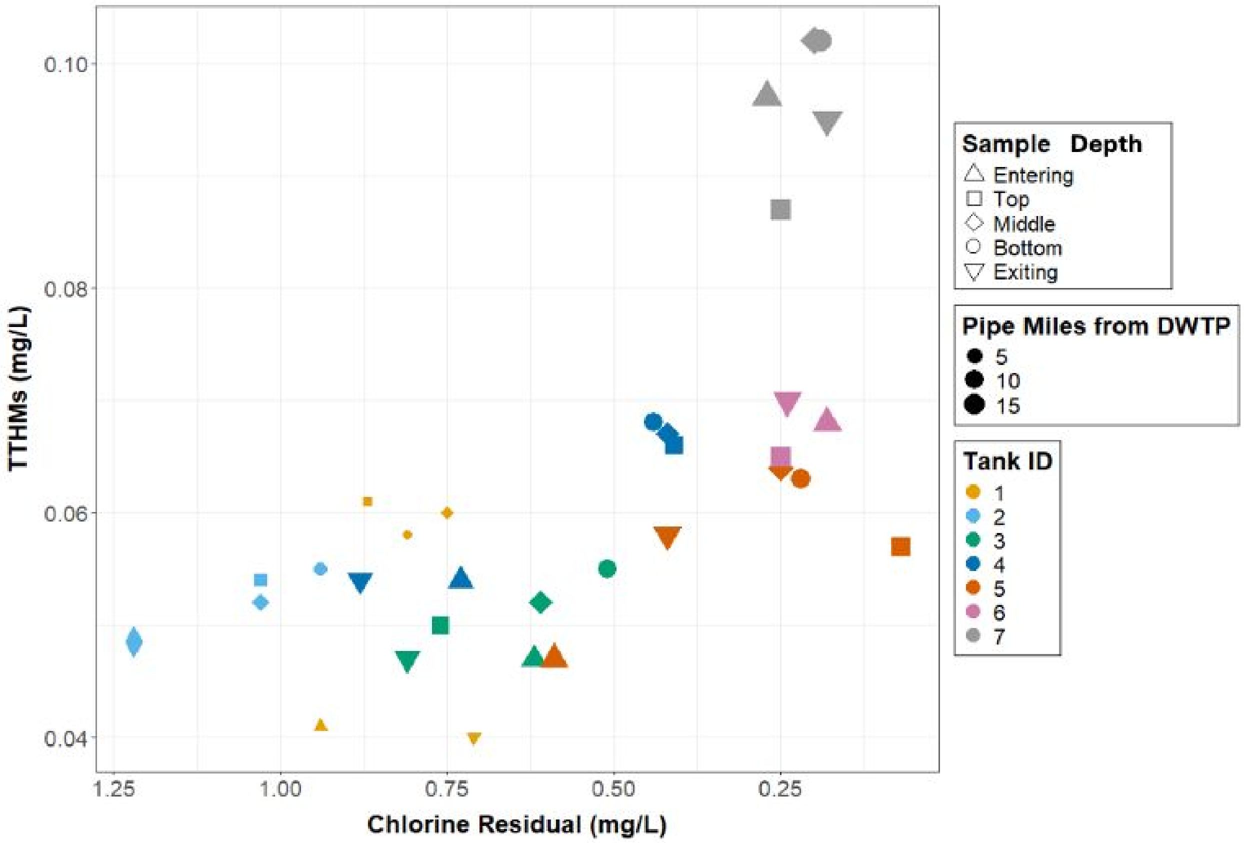

Figure S8: Chlorine residual vs TTHMs grouped by tank ID (i.e., site), sample depth within the tank, and pipe miles from the DWTP.

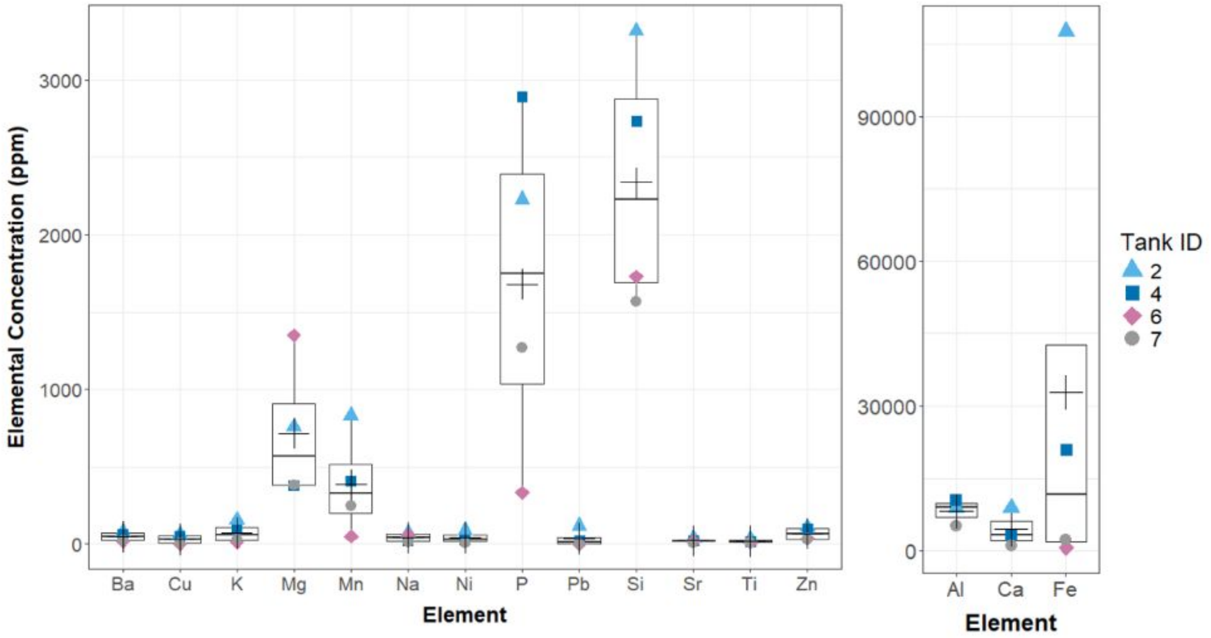

Figure S9: Elemental concentration of tank sediment.

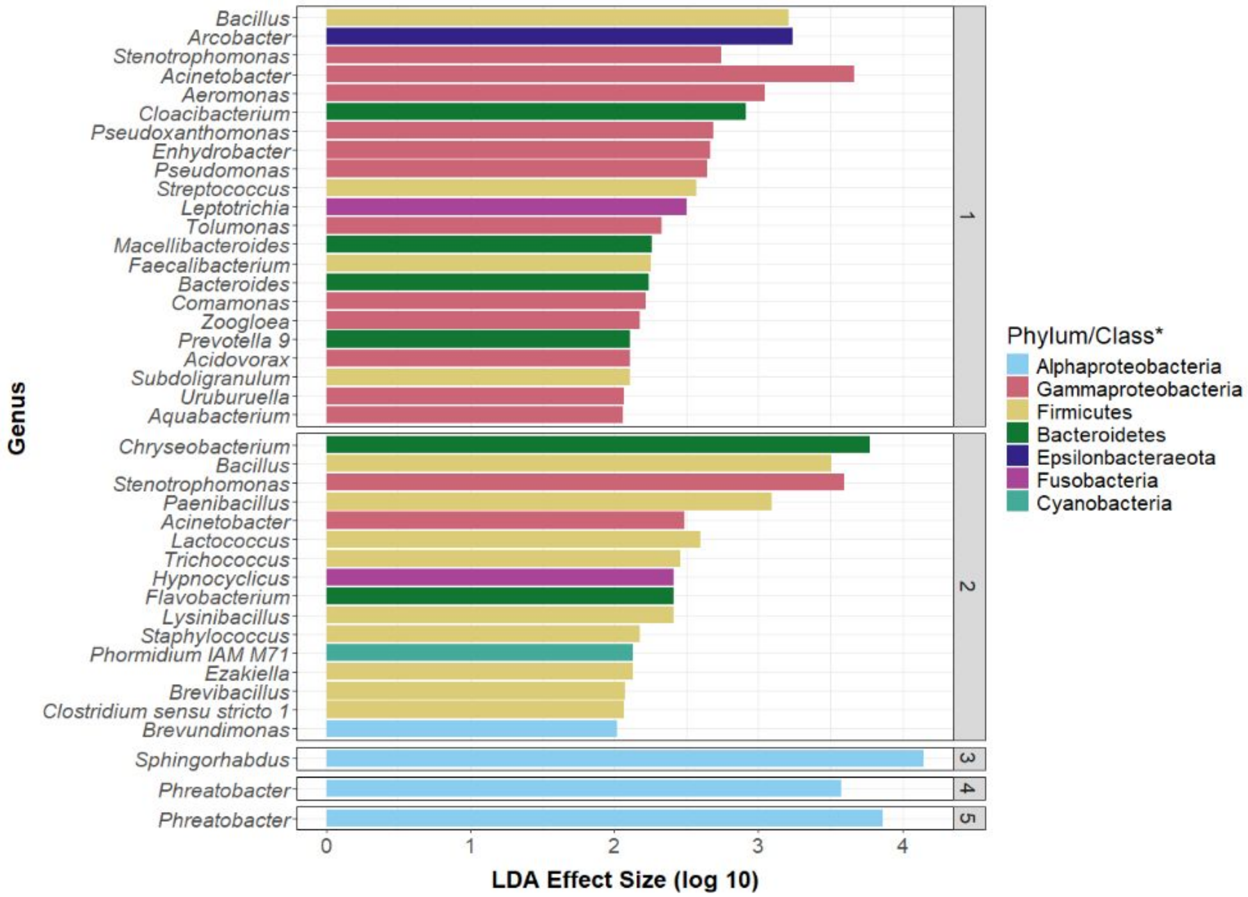

Figure S10: LDA effect size for genera between tank sites for water samples. Results shown are LDA > 2.5 and $p < 0.05$. Biomarkers with unknown or uncultured genera were excluded from the figure. Proteobacteria were split into class and all other taxa were identified at the phylum level.

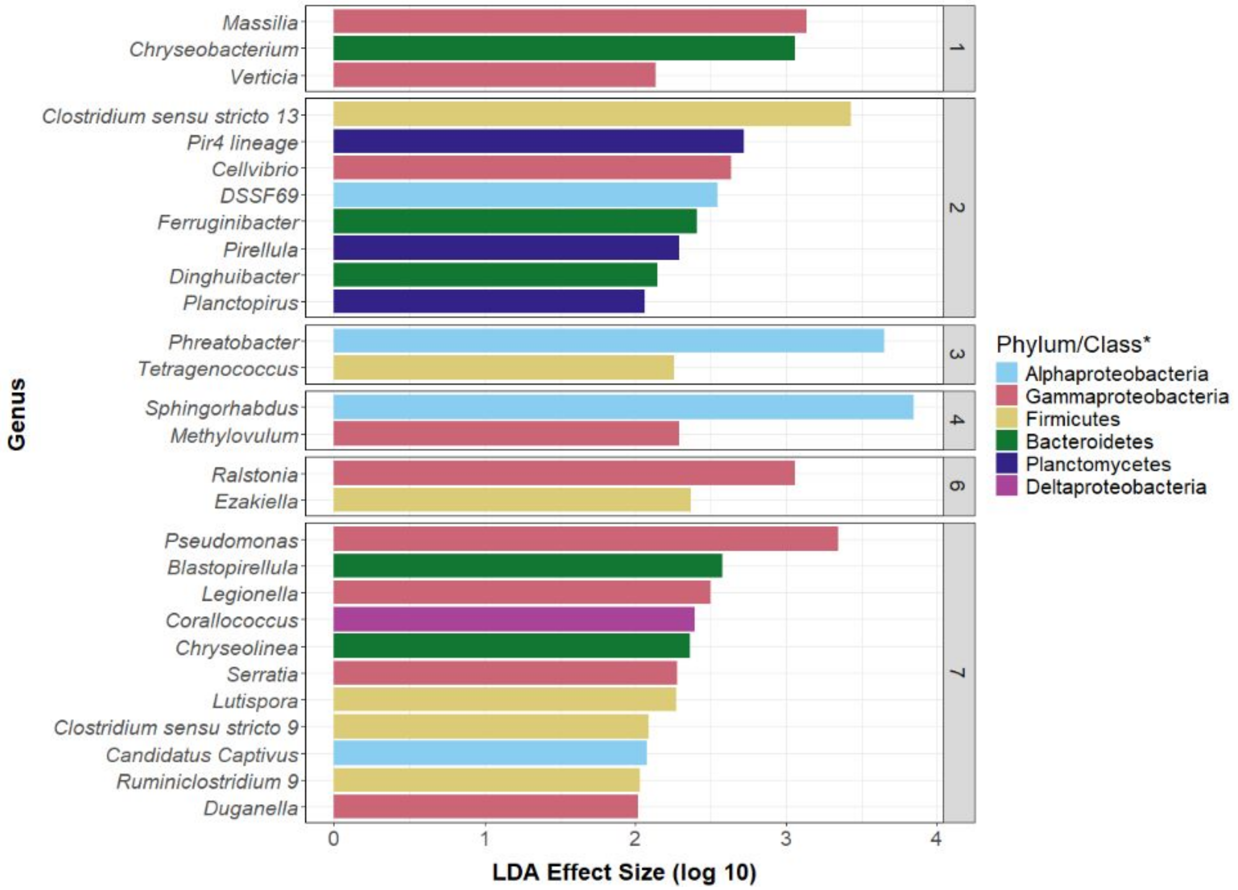

Figure S11: LDA effect size for genera between tank sites for sediment samples. Results shown are LDA > 2.5 and $p < 0.05$. Biomarkers with unknown or uncultured genera were excluded from the figure. Proteobacteria were split into class and all other taxa were identified at the phylum level.

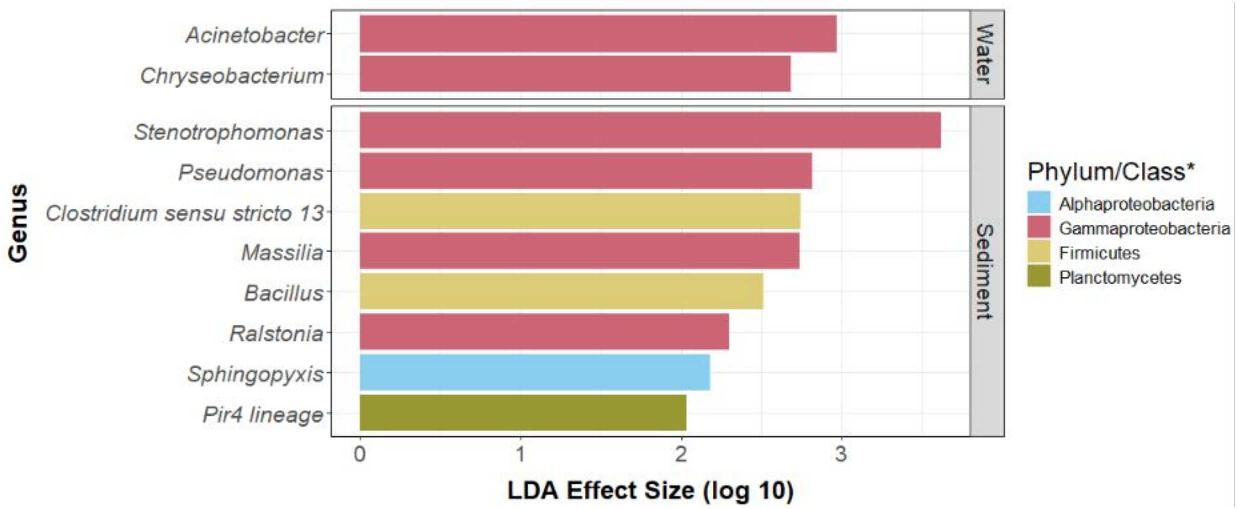

Figure S12: LDA effect size for genera between water and sediment. Results shown are LDA > 2.5 and $p < 0.05$. Biomarkers with unknown or uncultured genera were excluded from the figure. Proteobacteria were split into class and all other taxa were identified at the phylum level.

Table S4: Mantel test results of correlations between microbial community and distribution system characteristics. N = 33 for all parameters. Asterisks denote significance: *** $p \leq 0.001$, ** $p \leq 0.01$, * $p \leq 0.05$.

**Parameter**	**r**	**p-value**
**pH**	0.2624	0.0054**
Temperature	0.017	0.3648
**Conductivity**	0.213	0.002**
**TDS**	0.208	0.0021**
Turbidity	-0.006	0.4798
**Total chlorine**	0.473	0.0001***
Iron	-0.091	0.8478
Nitrite	0.139	0.0749
Nitrate	-0.024	0.5833
TTHMs	-0.024	0.5677
**TOC**	0.1939	0.0139*
**Pipe miles from treatment plant**	0.6613	0.0001***
**Tank turnover rate**	0.2671	0.0027**

Table S5: pH of each sample depth at each tank site.

**pH**						
	Entering	Top	Middle	Bottom	Exiting	Average
Tank 1	7.86	7.31	7.72	7.95	7.70	7.71
Tank 2	6.88	7.05	7.14	6.89	7.70	7.13
Tank 3	8.39	7.71	8.38	8.24	7.65	8.07
Tank 4	7.69	7.01	7.76	7.53	7.69	7.54
Tank 5	7.54	6.91	6.81	7.05	7.61	7.18
Tank 6	6.70	7.09	N/A	N/A	7.48	7.09
Tank 7	7.05	7.28	7.47	7.75	7.74	7.46

Table S6: Temperature (◦C) of each sample depth at each tank site.

**Temperature (◦C)**						
	Entering	Top	Middle	Bottom	Exiting	Average
Tank 1	23.4	21.7	22.1	22.4	22.9	22.5
Tank 2	22.3	23.0	24.0	24.5	25.4	23.8
Tank 3	21.2	21.2	21.8	22.2	22.6	21.8
Tank 4	21.5	22.1	22.3	23.6	22.0	22.3
Tank 5	19.7	21.1	20.2	19.8	19.9	20.1
Tank 6	24.5	25.1	N/A	N/A	23.9	24.5
Tank 7	21.6	22.6	22.2	22.1	21.8	22.1

Table S7: Conductivity (µs/cm) of each sample depth at each tank site.

**Conductivity (µs/cm)**						
	Entering	Top	Middle	Bottom	Exiting	Average
Tank 1	353	279	280	271	355	307.6
Tank 2	407	382	383	383	397	390.4
Tank 3	147.4	141.3	149.2	143.8	145.5	145.4
Tank 4	343	250	243	246	349	286.2
Tank 5	205.5	148.6	154.8	154.9	205.7	173.9
Tank 6	142.1	146.6	N/A	N/A	141.2	143.3
Tank 7	471	367	408	416	415	415.4

Table S8: Total dissolved solids (ppm) of each sample depth at each tank site.

**Total Dissolved Solids (ppm)**						
	Entering	Top	Middle	Bottom	Exiting	Average
Tank 1	246	194	194	190	247	214.2
Tank 2	281	267	266	268	276	271.6
Tank 3	98.8	102	105	104	111	104.2
Tank 4	241	177	170	174	246	201.6
Tank 5	140	108	106	106	139	119.8
Tank 6	103	104	N/A	N/A	102	103.0
Tank 7	322	253	280	289	287	286.2

Table S9: Total dissolved solids (ppm) of each sample depth at each tank site.

**Turbidity (NTU)**						
	Entering	Top	Middle	Bottom	Exiting	Average
Tank 1	0.00	0.04	0.00	2.37	0.00	0.482
Tank 2	0.18	0.05	0.05	0.17	0.02	0.094
Tank 3	0.67	0.66	0.88	0.63	0.72	0.712
Tank 4	0.01	0.10	0.13	0.18	4.11	0.906
Tank 5	0.00	0.08	0.02	0.03	0.10	0.046
Tank 6	0.13	0.65	N/A	N/A	0.43	0.403
Tank 7	0.00	0.01	0.05	0.03	0.06	0.030

Table S10: Total chlorine concentrations (mg/L) of each sample depth at each tank site.

**Total Chlorine (mg/L)**						
	Entering	Top	Middle	Bottom	Exiting	Average
Tank 1	0.94	0.87	0.75	0.81	0.71	0.82
Tank 2	1.22	1.03	1.03	0.94	1.22	1.09
Tank 3	0.62	0.76	0.61	0.51	0.81	0.66
Tank 4	0.73	0.41	0.42	0.44	0.88	0.58
Tank 5	0.59	0.07	0.25	0.22	0.42	0.31

Tank 6	0.18	0.25	N/A	N/A	0.24	0.22
Tank 7	0.27	0.25	0.20	0.19	0.18	0.22

Table S11: Iron concentrations (mg/L) of each sample depth at each tank site.

**Iron (mg/L)**						
	Entering	Top	Middle	Bottom	Exiting	Average
Tank 1	0.00	0.01	0.00	0.00	0.00	0.002
Tank 2	0.00	0.00	0.02	0.01	0.01	0.008
Tank 3	0.00	0.00	0.00	0.03	0.00	0.006
Tank 4	0.04	0.00	0.01	0.01	0.35	0.082
Tank 5	0.04	0.01	0.02	0.00	0.02	0.018
Tank 6	0.01	0.00	N/A	N/A	0.03	0.013
Tank 7	0.02	0.00	0.06	0.00	0.05	0.026

Table S12: Nitrite concentrations (mg/L) of each sample depth at each tank site.

**Nitrite (mg/L)**						
	Entering	Top	Middle	Bottom	Exiting	Average
Tank 1	0.004	0.004	0.001	0.001	0	0.0020
Tank 2	0	0	0.02	0.01	0.01	0.0080
Tank 3	0.000	0.005	0.004	0.003	0.004	0.0032
Tank 4	0.000	0.003	0.000	0.000	0.000	0.0006
Tank 5	0.003	0.001	0.000	0.003	0.000	0.0014
Tank 6	0.000	0.070	N/A	N/A	0.001	0.0237
Tank 7	0.000	0.001	0.002	0.003	0.006	0.0024

Table S13: Nitrate concentrations (mg/L as NO_3) of each sample depth at each tank site.

**Nitrate (mg/L as NO_3)**						
	Entering	Top	Middle	Bottom	Exiting	Average
Tank 1	3.96	8.36	5.28	1.76	6.60	5.192
Tank 2	7.04	6.16	7.04	4.84	6.16	6.248
Tank 3	6.60	5.72	1.32	4.40	4.84	4.576
Tank 4	6.16	3.08	5.72	4.84	3.08	4.576
Tank 5	5.72	4.40	3.52	3.52	3.08	4.048
Tank 6	9.68	4.84	N/A	N/A	6.16	6.893
Tank 7	4.84	7.48	7.04	7.04	2.20	5.720

Table S14: TTHM concentrations (mg/L) of each sample depth at each tank site.

**TTHMs (mg/L)**						
	Entering	Top	Middle	Bottom	Exiting	Average
Tank 1	0.041	0.061	0.06	0.058	0.04	0.0520
Tank 2	0.049	0.054	0.052	0.055	0.048	0.0516

Tank 3	0.047	0.05	0.052	0.055	0.047	0.0502
Tank 4	0.054	0.066	0.067	0.068	0.054	0.0618
Tank 5	0.047	0.057	0.064	0.063	0.058	0.0578
Tank 6	0.068	0.065	N/A	N/A	0.07	0.0677
Tank 7	0.097	0.087	0.102	0.102	0.095	0.0966

Table S15: Total organic carbon (mg/L) of each sample depth at each tank site.

**Total Organic Carbon (mg/L)**						
	Entering	Top	Middle	Bottom	Exiting	Average
Tank 1	2.039	1.866	1.819	2.192	2.359	2.055
Tank 2	2.490	0.437	2.414	2.539	2.524	2.081
Tank 3	2.141	2.119	1.503	2.070	2.190	2.005
Tank 4	2.123	1.975	1.920	2.060	2.197	2.055
Tank 5	1.829	2.029	1.803	2.012	1.871	1.909
Tank 6	1.823	1.956	N/A	N/A	1.797	1.859
Tank 7	2.810	2.429	2.345	2.416	2.324	2.465

Table S16: Protein, polysaccharide, and total EPS content.

**Tank ID**	**Replicate**	**Protein (mg/g)**	**Polysaccharide (mg/g)**	**Total EPS (mg/g)**
**1**	A	0.00587	0.01673	0.02259
	B	0.00587	0.02330	0.02917
**2**	A	0.00463	0.01804	0.02267
	B	0.00463	0.01640	0.02105
**3**	A	0.00373	0.02199	0.02572
	B	0.00389	0.01213	0.01602
**4**	A	0.00482	0.02001	0.02483
	B	0.00484	0.01607	0.02091
**6**	A	0.00187	0.00588	0.00775
	B	0.00171	0.00752	0.00923
**7**	A	0.00653	0.01870	0.02523
	B	0.00648	0.01640	0.02288

Table S17: R^2 and efficiency values.

**Run #**	**Tank and sample depth**	**R^2**	**Efficiency**
Run 1	Tank 3 entering	0.995	77.5
	Tank 3 top		
	Tank 3 middle		
	Tank 3 bottom		
	Tank 3 exiting		
	Tank 5 sediment A		
	Tank 5 sediment B		

	Tank 7 sediment A		
	Tank 7 sediment B		
Run 2	Tank 1 entering	0.988	80.4
	Tank 1 top		
	Tank 1 middle		
	Tank 1 bottom		
	Tank 1 exiting		
	Tank 2 entering		
	Tank 2 top		
	Tank 2 middle		
	Tank 2 bottom		
	Tank 4 entering		
	Tank 4 top		
	Tank 4 middle		
	Tank 4 bottom		
	Tank 4 exiting		
	Tank 6 entering		
	Tank 6 top		
	Tank 6 exiting		
Run 3	Tank 5 entering	0.988	74.2
	Tank 5 middle		
	Tank 5 bottom		
	Tank 5 exiting		
	Tank 7 entering		
	Tank 7 top		
	Tank 7 middle		
	Tank 7 exiting		
Run 4	Tank 5 top	0.979	76.3
	Tank 7 bottom		
Run 5	Tank 2 exiting	0.994	60.7
	Tank 2 sediment A		
	Tank 2 sediment B		
	Tank 4 sediment A		
	Tank 4 sediment B		
	Tank 6 sediment A		
	Tank 6 sediment B		
Run 6	Tank 5 sediment A	0.999	65.5
	Tank 7 sediment A		
	Tank 7 sediment B		
Run 7	Tank 1 sediment A	0.927	85.9
	Tank 1 sediment B		
	Tank 3 sediment A		
	Tank 3 sediment B		

References

(1) Standard Methods Committee of the American Public Health Association; American Water Works Association; Water Environment Federation. 9223 Enzyme Substrate Coliform Test. In *Standard Methods For the Examination of Water and Wastewater*; Standard Methods for the Examination of Water and Wastewater; American Public Health Association, 2017. https://doi.org/10.2105/SMWW.2882.194.

(2) Suzuki, M. T.; Taylor, L. T.; DeLong, E. F. Quantitative Analysis of Small-Subunit rRNA Genes in Mixed Microbial Populations via 5′-Nuclease Assays. *Appl. Environ. Microbiol.* **2000**, *66* (11), 4605–4614. https://doi.org/10.1128/AEM.66.11.4605-4614.2000.

(3) Elhadidy, A. M.; Dyke, M. I. V.; Chen, F.; Peldszus, S.; Huck, P. M. Development and Application of an Improved Protocol to Characterize Biofilms in Biologically Active Drinking Water Filters. *Environ. Sci. Water Res. Technol.* **2017**, *3* (2), 249–261. https://doi.org/10.1039/C6EW00279J.

(4) Frølund, B.; Palmgren, R.; Keiding, K.; Nielsen, P. H. Extraction of Extracellular Polymers from Activated Sludge Using a Cation Exchange Resin. *Water Res.* **1996**, *30* (8), 1749–1758. https://doi.org/10.1016/0043-1354(95)00323-1.

(5) Siddharth, T.; Sridhar, P.; Vinila, V.; Tyagi, R. D. Environmental Applications of Microbial Extracellular Polymeric Substance (EPS): A Review. *J. Environ. Manage.* **2021**, *287*, 112307. https://doi.org/10.1016/j.jenvman.2021.112307.

(6) O'Callaghan, J. P.; White, A. Pierce BCA Protein Assay Protocol. **2025**. https://doi.org/dx.doi.org/10.17504/protocols.io.x54v9rod4v3e/v1.

(7) DuBois, Michel.; Gilles, K. A.; Hamilton, J. K.; Rebers, P. A.; Smith, Fred. Colorimetric Method for Determination of Sugars and Related Substances. *Anal. Chem.* **1956**, *28* (3), 350–356. https://doi.org/10.1021/ac60111a017.

(8) Khleborodova, A.; Gamboa-Tuz, S. D.; Ramos, M.; Segata, N.; Waldron, L.; Oh, S. Lefser: Implementation of Metagenomic Biomarker Discovery Tool, LEfSe, in R. *Bioinformatics* **2024**, *40* (12), btae707. https://doi.org/10.1093/bioinformatics/btae707.

(9) Falkinham, J. O. Living with Legionella and Other Waterborne Pathogens. *Microorganisms* **2020**, *8* (12), 2026. https://doi.org/10.3390/microorganisms8122026.

(10) Karumathil, D. P.; Yin, H.-B.; Kollanoor-Johny, A.; Venkitanarayanan, K. Effect of Chlorine Exposure on the Survival and Antibiotic Gene Expression of Multidrug Resistant Acinetobacter Baumannii in Water. *Int. J. Environ. Res. Public. Health* **2014**, *11* (2), 1844–1854. https://doi.org/10.3390/ijerph110201844.
